# Coixenolide Enhances Antitumor Immunity of Cytotoxic CD8^+^ T Cells by Inhibiting S100A8/A9‐CD36 Axis in Obesity‐Associated Pancreatic Adenocarcinoma

**DOI:** 10.1002/advs.202515759

**Published:** 2026-08-25

**Authors:** Kaidi Chen, Rong Chen, Wei Li, Bin Song, Ruiying Zhang, Lewei Dai, Yuwei Qi, Ruqian Chen, Yifan Xu, Hangbin Li, Jingyi Huang, Fangping Wu, Jianan Peng, Xinyu Wang, Yang Xiong, Chaofeng Mu, Guilin Cheng, Zhaoshen Li, Shanming Ruan, Lei Huang, Mancang Gu

**Affiliations:** ^1^ Jinhua Academy of Zhejiang Chinese Medical University, Zhejiang Chinese Medical University Jinhua Zhejiang People's Republic of China; ^2^ School of Pharmaceutical Sciences Zhejiang Chinese Medical University Hangzhou Zhejiang People's Republic of China; ^3^ Academy of Chinese Medical Sciences Zhejiang Chinese Medical University Hangzhou Zhejiang People's Republic of China; ^4^ Department of Hepatobiliary and Pancreatic Surgery, Changhai Hospital Naval Medical University Shanghai People's Republic of China; ^5^ School of Basic Medical Sciences Guangzhou University of Chinese Medicine Guangzhou People's Republic of China; ^6^ National Key Laboratory of Immunity and Inflammation National Clinical Research Center for Digestive Diseases Shanghai Institute of Pancreatic Diseases, Changhai Clinical Research Unit, Department of Gastroenterology, The First Affiliated Hospital of Naval Medical University / Changhai Hospital Naval Medical University Shanghai People's Republic of China; ^7^ Department of Medical Oncology The First Affiliated Hospital of Zhejiang Chinese Medical University (Zhejiang Provincial Hospital of Traditional Chinese Medicine) Hangzhou Zhejiang People's Republic of China; ^8^ Institute For Innovation in Modern Traditional Chinese Medicine The First Affiliated Hospital of Zhejiang Chinese Medical University (Zhejiang Provincial Hospital of Traditional Chinese Medicine) Hangzhou Zhejiang People's Republic of China

**Keywords:** CD8^+^ T cells, coixenolide, ferroptosis, high‐fat diet, lipid peroxidation, obesity‐associated pancreatic adenocarcinoma, S100A8/A9‐CD36 axis, tumor microenvironment‐based immunotherapy

## Abstract

Pancreatic adenocarcinoma (PAAD), characterized by an immunosuppressive tumor microenvironment (TME) and resistance to immunotherapy, exhibits a dismal prognosis further exacerbated by obesity. This study demonstrates the potent immunomodulatory and antitumor effects of coixenolide, a bioactive fatty acid ester from Coix lacryma‐jobi L., particularly in obesity‐associated PAAD. Utilizing high‐fat diet (HFD)‐fed mouse models, we found that coixenolide significantly inhibited tumor growth in HFD mice compared to control diet (CD) mice, correlating with enhanced intra‐tumoral CD8^+^ T cell infiltration and cytotoxicity, reduced regulatory T cells (Tregs), and increased tumor cell apoptosis. Single‐cell RNA sequencing (scRNA‐seq) of PAAD tissues revealed that coixenolide specifically expanded and activated cytotoxic CD8^+^ T cells within the TME of HFD mice. Further analysis identified distinct malignant epithelial cell clusters enriched in HFD tumors, characterized by high expression of *S100a8/a9* and stemness potential. Coixenolide treatment significantly reduced these *S100a8/a9*‐expressing tumor cell populations and suppressed *S100a8/a9* protein expression and secretion. Cell–cell communication analysis and functional assays revealed that tumor cell‐derived *S100a8/a9* interacted with *Cd36* on CD8^+^ T cells. This interaction promoted CD36‐mediated fatty acid uptake, leading to lipid peroxidation, ferroptosis, and functional exhaustion of CD8^+^ T cells. Coixenolide disrupted this axis by inhibiting tumor cell *S100a8/a9*, thereby downregulating CD36 on CD8^+^ T cells, reducing lipid peroxidation and ferroptosis, and restoring CD8^+^ T cell cytotoxicity. Genetic knockout (*S100a9* KO) of tumor cells mimicked coixenolide's effects, enhancing CD8^+^ T cell function and suppressing tumor growth in vivo, especially in HFD mice. Clinical data confirmed elevated S100A8/A9 expression in human PAAD tumors correlated with poor survival, advanced stage, and higher histological grade, and scRNA‐seq of patient samples showed S100A8/A9 expression in malignant ductal cells. Collectively, these results identify coixenolide as an immune‐metabolic modulator that attenuates obesity‐driven immunosuppression in PAAD at least in significant part through inhibition of the S100A8/A9‐CD36 axis, with lipid peroxidation and ferroptosis of CD8^+^ T cells reduced. Other mechanisms potentially involving regulatory T cells warrant further investigation in the future. These findings suggest S100A8/A9‐CD36‐mediated intercellular crosstalk as a therapeutically actionable immune‐metabolic vulnerability in obesity‐associated PAAD.

## Graphical abstract

Pancreatic adenocarcinoma (PAAD) remains highly lethal with limited treatment options. This study demonstrates that coixenolide, a bioactive compound from Coix lacryma‐jobi L. and a key component of the clinically approved Kanglaite injection, exhibits enhanced antitumor efficacy in high‐fat diet (HFD)‐induced obese mice bearing PAAD tumors compared to control diet (CD)‐fed mice. Using single‐cell RNA sequencing and functional validation, we reveal that coixenolide selectively promotes infiltration and activation of cytotoxic CD8^+^ T cells accompanied by reduced Treg infiltration specifically in the obese tumor microenvironment. Mechanistically, coixenolide targets S100A8/A9‐expressing tumor cell subpopulations that exhibit high stemness and are enriched in obese conditions. This inhibition disrupts S100A8/A9‐CD36‐mediated crosstalk between tumor cells and CD8^+^ T cells, thereby reducing CD36‐dependent fatty acid uptake, lipid peroxidation, and ferroptosis in CD8^+^ T cells. Consequently, CD8^+^ T cells show enhanced cytokine production (TNF‐α, GZMB) and cytotoxicity. Genetic ablation of *S100a9* in tumor cells recapitulates coixenolide's effects, supporting this axis as a major contributor for restoring antitumor immunity. Clinically, high S100A8/A9 expression correlates with poor survival in PAAD patients. Our findings establish coixenolide as a novel immune‐metabolic modulator that overcomes obesity‐driven CD8^+^ T cell dysfunction at least in significant part by targeting the S100A8/A9‐CD36 pathway, providing a strategic foundation for immunotherapy‐resistant PAAD, particularly obesity‐associated subtypes.

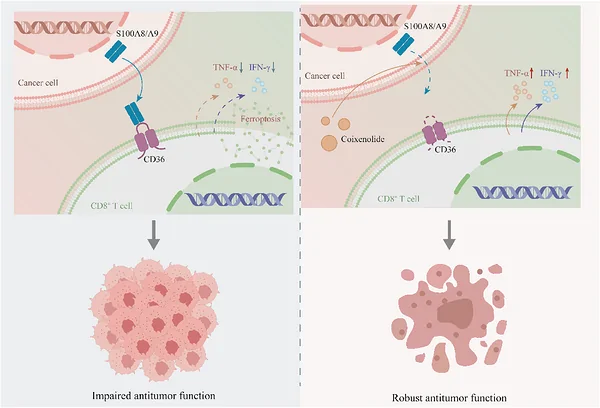

## 1 Introduction

Pancreatic adenocarcinoma (PAAD) is among the most fatal malignancies, exhibiting a 5‐year overall survival rate of less than 8% worldwide [1]. It stood as the sixth leading cause of cancer‐associated deaths in 2022 globally [2], and is expected to rank second by 2030 [3, 4]. For PAAD, therapeutic modalities are severely lacking currently, and surgery remains the only potential cure for a limited portion of nonmetastatic diseases [5, 6]. It is highly difficult to diagnose PAAD early, often leading to missed surgical opportunities. Moreover, combination chemotherapies such as FOLFIRINOX and gemcitabine/nab‐paclitaxel are limited by severe toxic adverse events, with largely limited improvement in the 5‐year survival rate [7, 8]. There also lack effective targeted therapy and immunotherapy against PAAD.

The emergence of immunotherapy has opened new avenues for the treatment of PAAD, with T cells playing a pivotal role in the immune defense against cancer [9]. Specifically, among T cell subsets, cytotoxic CD8^+^ T cells are capable of directly eliminating tumor cells [10]. However, the TME of PAAD is immunologically “cold”, which is composed of abundant stroma and is enriched with immunosuppressive cells that support the survival of tumor cells by various cell‐to‐cell communication pathways [11]. The admixture of suppressive cells with cytotoxic CD8^+^ T cells importantly facilitates immune tolerance and tumor evasion from immunologic clearance [12, 13, 14]. Therefore, PAAD is considered one of the most immunotherapy‐resistant cancer types [15]. In light of this, increasing evidence suggests that obesity is also a significant factor impacting anti‐PAAD immunity. In recent years, the proportion of patients with PAAD who are obese at the commencement of therapy has been rapidly increasing. Obesity not only elevates the risk of PAAD but also promotes chronic inflammation and T cell dysfunction, thereby facilitating the initiation and progression of PAAD [16]. Obesity‐induced changes in systemic metabolism can affect the interactions between immune cells and cancer cells while creating an inflammatory and immunosuppressive TME [17, 18]. Therefore, interventions to support and enhance antitumor immunity are critical for effective immunotherapy against PAAD, especially obesity‐associated PAAD.

Research on Chinese herbal medicine extensively explores its immune regulation activities [19]. Coixenolide (CAS: 29066‐43‐1) is a bioactive fatty acid ester extracted from Coix lacryma‐jobi L. (Coix agrestis Lour.), and has been used for rheumatism, neuralgia, diuretic medications, and anticancer treatment [20]. Moreover, coixenolide is one of the principal components of a patent Chinese medicine injection, Kanglaite injection (KLTi), which was approved by the China Food and Drug Administration in 2010 (approval number: Z10970091). The clinical cancer indications of coixenolide are mainly non‐small‐cell lung cancer and primary liver cancer [21, 22, 23]. In 2014, a US phase II clinical trial of KLTi for advanced pancreatic cancer [24] revealed that coixenolide can overcome chemoresistance, increase treatment tolerance, and significantly improve survival and quality of life of patients when combined with gemcitabine. A recent study [25] confirmed the properties of coixenolide in regulating the tumor immune microenvironment and promoting antitumor immune responses. However, the specific mechanisms underlying how coixenolide regulates the immune microenvironment in obesity‐associated PAAD are not yet fully understood.

Herein, we demonstrated the immunomodulatory effect of coixenolide on obese cases with PAAD. Through single‐cell transcriptomic sequencing (scRNA‐seq), we elucidated the mechanism via which coixenolide inhibited PAAD cells and activated cytotoxic CD8^+^ T cells, and specifically identified the involved ligand–receptor interactions. Investigating the effect of coixenolide on PAAD and the underlying mechanism could enrich our understanding of immune modulation in anticancer therapy, facilitate the development of enhanced anticancer therapies utilizing coixenolide, and help establish novel strategies for anticancer therapy targeting the interplay between tumor cells and immune cells in PAAD.

## 2 Results

### 2.1 Coixenolide Promoted T Cell‐Mediated Tumor Cell Killing in High Fat Diet (HFD)‐Fed Mice

To model human obesity, we randomized C57BL/6J mice at five weeks of age to control diet (CD) and HFD groups fed ad libitum (Figure 1A) [17]. 10% and 60% of the kilocalories in the CD and HFD chow came from fat (saturated and unsaturated), respectively. After 8 weeks of feeding, mice in the HFD group gained significantly more weight (Figure S1A) and exhibited obesity‐associated systemic metabolic changes, including increased levels of blood glucose, cholesterol, and triglycerides (Figure S1B–D). Anatomical analyses revealed a significant accumulation of adipose tissue in the dorsal region of mice in the HFD group (Figure 1B). After adaptation to CD or HFD, mice were transplanted with Pan02 cells, and then the experimental tumor‐bearing mice received intraperitoneal injections of coixenolide (2.50 or 1.25 g/kg/day) for 14 consecutive days since the tumors became palpable. In the control group mice, we administered an equal volume of empty vehicle without coixenolide. Utilizing the IVIS imaging system, a dose‐dependent decrease in the relative bioluminescent intensity of tumors was observed in subjects treated with increasing concentrations of coixenolide in both the CD and HFD groups (Figure 1C,D). In the CD‐fed mice, the tumor growth inhibition rates (TGIs) in the low‐ and high‐coixenolide concentration groups were 24% and 35%, respectively, while in the HFD‐fed mice, the TGIs in the low‐ and high‐coixenolide concentration groups were 45% and 70%, respectively (Figure 1E,F). These suggest that coixenolide exhibits more pronounced therapeutic efficacy against tumors of HFD‐fed mice compared to those of CD‐fed mice. Tumor tissues from each group were collected at the end of the experiments. Oil Red O staining revealed substantial infiltration of adipocytes in the tumor samples of HFD‐fed mice (Figure S1G). To further dissect the driving forces of restricted tumor growth under coixenolide treatment, we performed immunocytochemistry to determine the proliferation and apoptosis of tumor cells. Notably, coixenolide treatment prominently diminished tumor cell proliferation (as detected by Ki‐67 staining), and significantly increased cell apoptosis (as assessed by TUNEL staining) (Figure 1F,G); the effects were also more pronounced in the HFD group than in the CD group. In both the HFD‐ and CD‐fed mice, there were no significant changes in the average body weight of mice in the coixenolide group compared with the vehicle control group during the treatment period, confirming the in vivo safety of coixenolide (Figure S1H,I).

**FIGURE 1:**
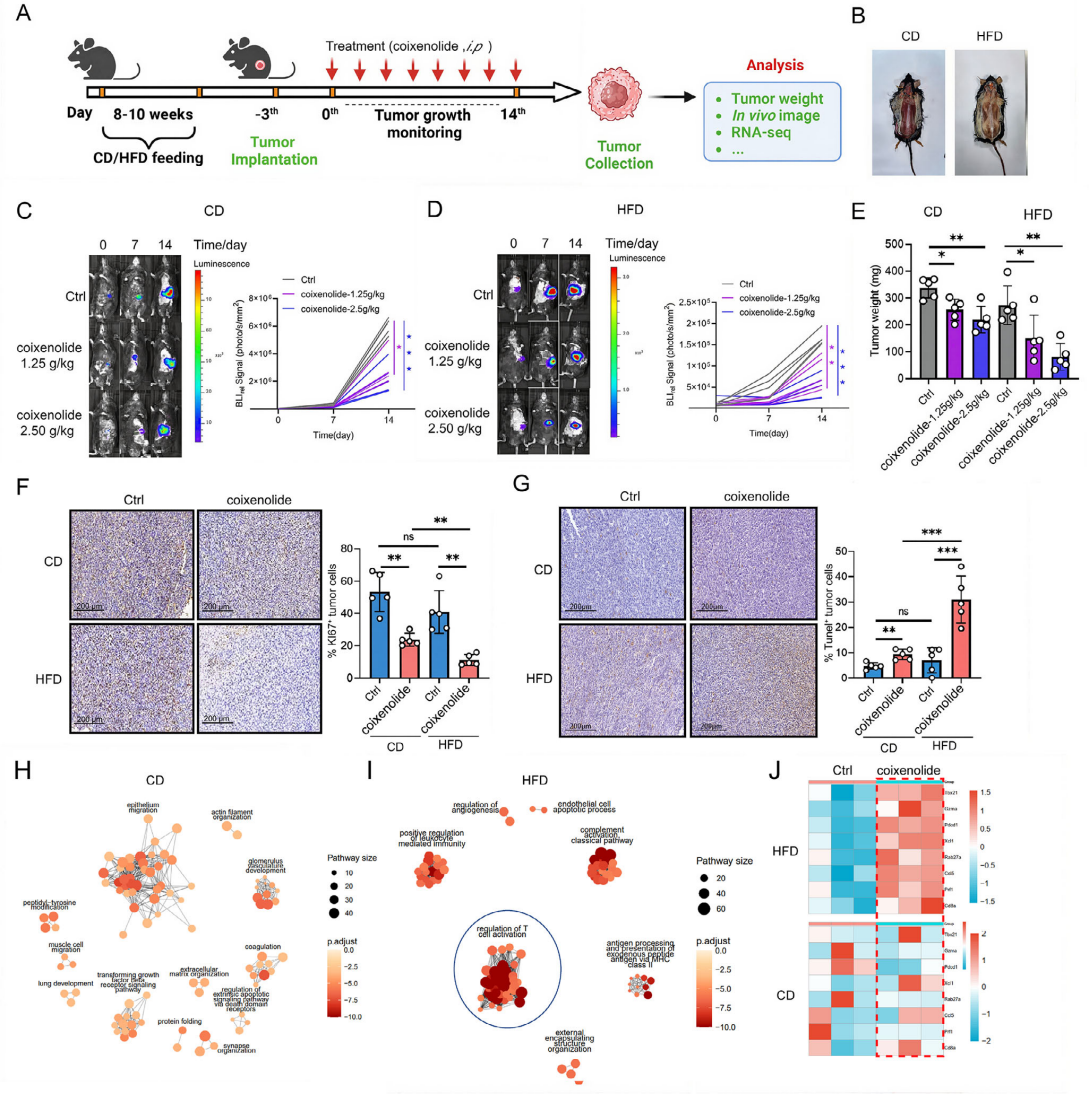
Coixenolide enhanced the ability of CD8^+^ T cells to kill PAAD cells in HFD‐fed mice. (A) Schematic diagram depicting the experimental setup. (B) Anatomy of the dorsal surface of CD‐ or HFD‐fed mice. (C,D) In vivo imaging of pancreatic tumors of CD‐ (C) or HFD‐fed (D) mice treated with coixenolide (1.25 or 2.5 g/kg) or vehicle control. (E) The tumor weights of CD‐ or HFD‐fed mice treated with coixenolide (1.25 or 2.5 g/kg) or vehicle control. *n* = 5 mice per group. Data are presented as mean ± SD. Statistical significance was determined by two‐way ANOVA followed by Tukey's post‐hoc test. All comparisons are two‐sided. (F,G) Immunohistochemical staining showing Ki‐67^+^ (F) or TUNEL^+^ (G) cells in tumors of the CD and HFD groups treated with coixenolide (2.5 g/kg) or vehicle control. *n* = 5 biologically independent samples per group. Data are mean ± SD; two‐sided Student's *t*‐test was used for pairwise comparisons. Scale bars, 200 µm. (H,I) The enriched pathways associated with DEGs in tumors of the CD (H) and HFD (I) groups following coixenolide treatment. (J) The expression levels of activation and cytotoxicity genes of CD8^+^ T cells in tumors of the HFD and CD groups treated with coixenolide versus control. *n* = 3 per group. ns, not significant; *p < 0.05, **p < 0.01, ***p < 0.001.

We further performed high‐throughput sequencing to investigate the impact of coixenolide on antitumor immunity in both the HFD and CD groups. In tumors of the HFD group, we observed significant upregulation of 596 genes and downregulation of 551 genes following coixenolide treatment, compared to the vehicle control (Figure S1J). Moreover, coixenolide significantly upregulated the expression of 969 genes and downregulated the expression of 481 genes in tumors of the CD group (Figure S1K). However, only 76 differentially expressed genes (DEGs) were shared between the HFD and CD groups, suggesting the potentially varying mechanisms of action of coixenolide in different groups (Figure S1L). We further performed gene ontology (GO) biological process annotation to study the function of the DEGs, which exhibited a predominant enrichment in cancer‐associated pathways in tumors of the CD group (Figure 1H). In tumors of the HFD group, in addition to being associated with cancer‐related pathways, the DEGs were mainly involved in lymphocyte‐mediated immunity, especially T cell activation pathways (Figure 1I). These indicated that coixenolide specifically activated T cell‐mediated immune responses in tumors of HFD‐fed mice, exerting an enhanced therapeutic effect.

To further investigate the impact of coixenolide on T cells, we initially employed seven distinct immune cell infiltration algorithms to infer the potential changes in T cells following coixenolide administration. In the TME of HFD‐fed mice, coixenolide‐treated groups displayed higher proportions of CD8^+^ T cells compared to the control group. However, the proportion of CD8^+^ T cells in tumors of CD‐fed mice did not show a significant change after coixenolide treatment. No significant alterations were observed in naive CD4^+^ T cells, resting memory CD4^+^ T cells, activated memory CD4^+^ T cells, T follicular helper cells, regulatory T cells (Tregs), or others, indicating that CD8^+^ T cells might serve as the crucial effector cells associated with the immunomodulatory effects of coixenolide (Figure S1M,N). Moreover, coixenolide upregulated the cytotoxicity markers of CD8^+^ T cells in HFD‐fed mice rather than CD‐fed mice (Figure 1J).

### 2.2 Single‑Cell Transcriptomics Atlas of the Heterogeneous Tumor Microenvironment in Coixenolide‐Treated Mice

To further delineate the cellular landscape of PAAD subjected to coixenolide treatment, we performed scRNA‐seq evaluation of 13 pancreatic cancer specimens, including 7 tissues obtained from the HFD group and 6 from the CD group (Figure 2A). We annotated a total of 85 360 cells after filtering out cells with low RNA content or high mitochondrial RNA. These included 25 145 and 13 705 cells originating from the HFD and CD groups, respectively, following coixenolide treatment, and concurrently, 21 334 and 25 176 cells obtained from the control arms of the HFD‐ and CD‐fed mice, respectively (Figure 2B,C).

**FIGURE 2:**
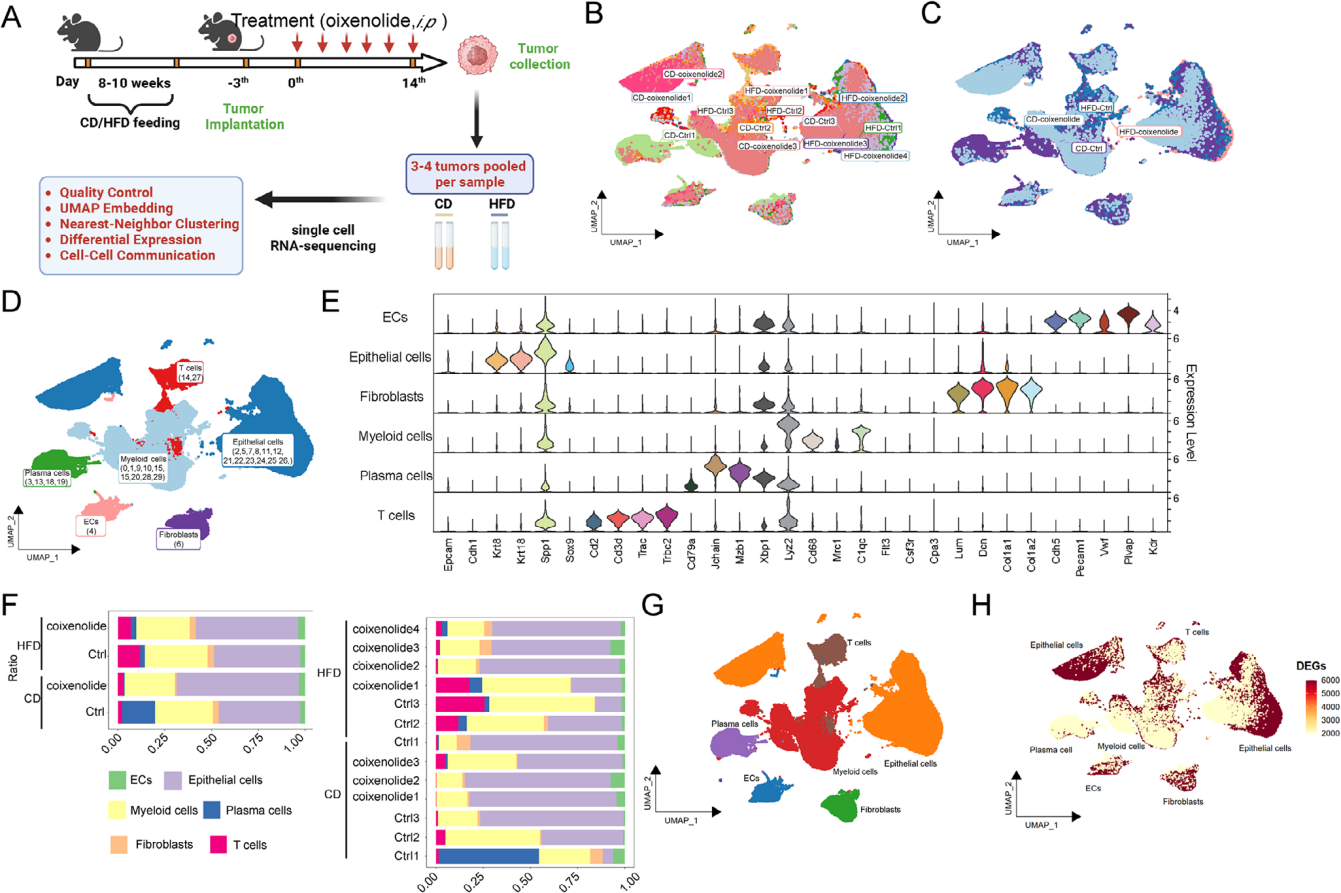
Single‐cell transcriptomic analysis of the PAAD tissues in HFD‐ and CD‐ fed mice. (A) Schematic diagram depicting the single‐cell RNA‐seq experiment and analysis. (B–D) The integrated cell map, which consists of 13 tissue samples (B), 4 groups (C), and 30 clusters (D) from 6 annotated cell types. (E) Representative marker genes across cell types. (F) The heterogeneity of cell types among different groups and tissue samples based on cell ratio. (G,H) The distribution of major cell types (G) and the number of differentially expressed genes in each cell type (H).

Unsupervised clustering of gene expression revealed 30 clusters (Figure 2D). The cells were classified into six types based on previously well‐defined gene markers: Epithelial cells (*n* = 43 461) were identified by high expressions of *Epcam*, *Cdh1*, *Krt8*, *Krt18*, *Spp1*, and *Sox9*; T cells (*n* = 5189) expressed *Cd2*, *Cd3d*, *Trac*, and *Trbc2*; plasma cells (*n* = 5819) were identified by *Cd79a*, *Jchain*, *Mzb1*, and *Xbp1*; myeloid cells (*n* = 25,823) had high expressions of *Lyz2*, *Cd68*, *Mrc1*, *C1qc*, *Flt3*, *Csf3r*, and *Cpa3*; fibroblasts (*n* = 2455) were marked by *Lum*, *Dcn*, *Col1a1*, and *Col1a2*; and endothelial cells (*n* = 2613) exhibited high expressions of *Cdh5*, *Pecam1*, *Vwf*, *Plvap*, and *Kdr* (Figure 2E) [26, 27]. The infiltration of the six cell types varied across different samples, albeit with epithelial cells being the most abundant and constituting approximately half of the total cell population (Figure 2F). Notably, epithelial cells also harbored the most DEGs after coixenolide treatment (Figure 2G,H). Although myeloid cells also had high infiltration levels in all samples, there was no significant difference across different groups. Overall, T cell infiltration was relatively low; however, there was an obvious change in the abundance of T cells after coixenolide administration (Figure 2F).

### 2.3 Coixenolide Enhanced CD8^+^ T Cell Infiltration and Cytotoxicity in HFD‐Fed Mice With PAAD

Considering the potential of coixenolide in activating anti‐PAAD immune responses in HFD‐fed mice, our subsequent objective was to delineate the impact of coixenolide on the immunological alterations within tumors. T cells were the predominant subset of infiltrating lymphocytes in the TME of PAAD; therefore, we further explored their subsets by performing second‐level clustering analyses. In the Uniform Manifold Approximation and Projection (UMAP) plot analysis of the scRNA‐seq data, types of T cells in the tumors were assessed by their gene expression signatures. A total of six subpopulations of T cells were identified, including Treg, cytotoxic CD8^+^ T, Tfh, CD8^+^ Tex, NKT, and GDT cells (Figure 3A–C).

**FIGURE 3:**
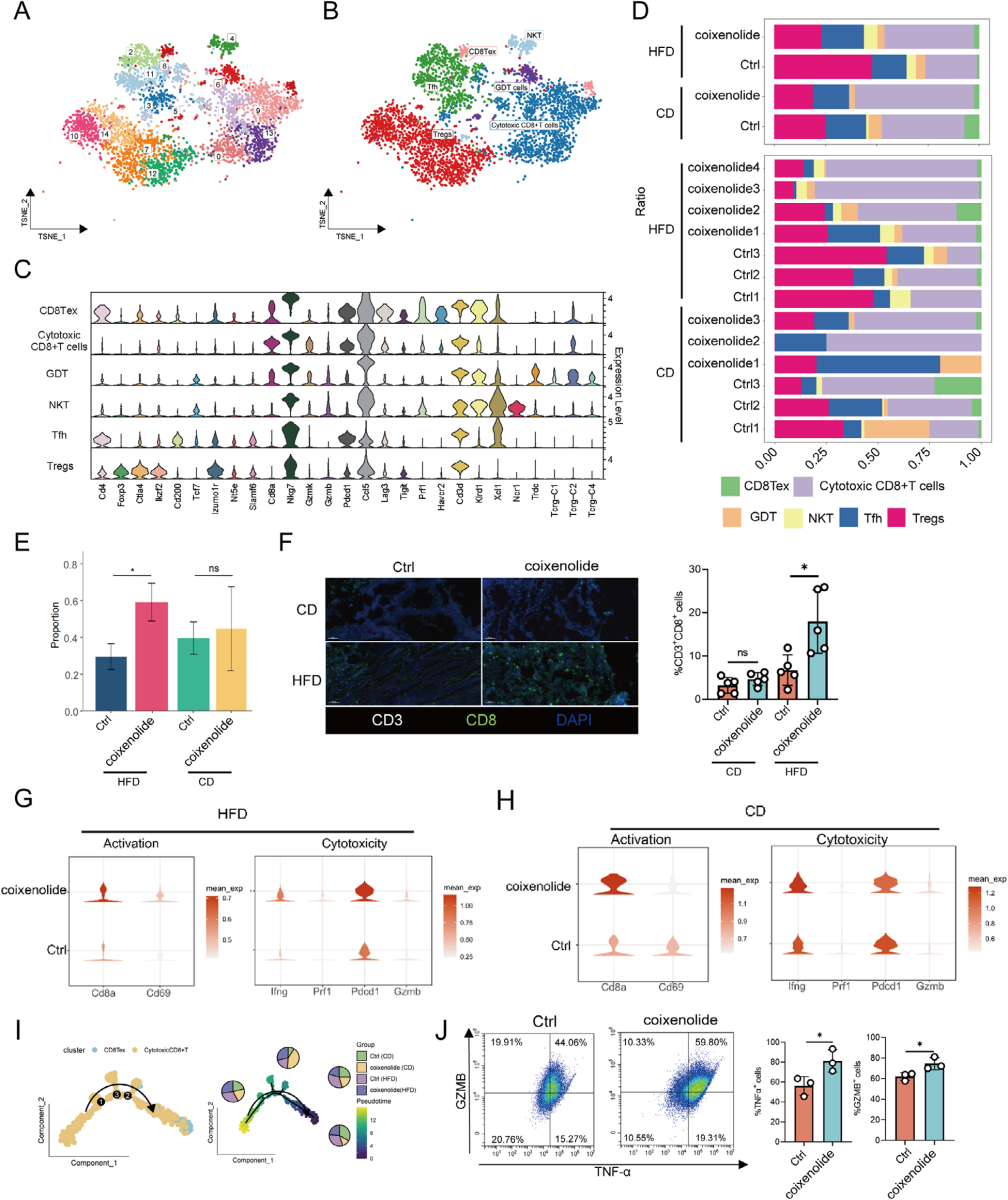
Transcriptomic analyses revealed that coixenolide significantly activated CD8^+^ T cells in the tumor microenvironment of high‐fat diet (HFD)‐fed mice. (A,B) UMAP plots showing 15 clusters (A) from 6 annotated T cell subtypes (B). (C) The representative marker genes across T cell types. (D) The heterogeneity of T cell types among different groups and tissue samples based on cell ratio. (E) Changes in the proportion of cytotoxic CD8^+^ T cells within the tumor microenvironment of HFD‐ and CD‐fed mice following coixenolide administration. *n* = 7 for HFD group and *n* = 6 for CD group. Data are mean ± SD; a two‐sided *t*‐test was used. (F) Immunofluorescent staining showing co‐localization of CD3 (white), CD8 (green), and DAPI (deep blue) in the tissues of CD‐ and HFD‐fed mice following coixenolide administration; representative of three independent experiments. Scale bars, 100 µm. (G,H) Expression levels of activation, cytotoxicity, and inhibitory receptor genes in cytotoxic CD8^+^ T cells of the tumors in HFD (G) and CD (H)‐fed mice following coixenolide treatment. *n* = 3 per group. (I) Semi‐supervised pseudo‐time trajectory of CD8^+^ T cell subtypes using Monocle2. The trajectory is colored by cell types and sample groups. (J) Flow cytometry plot measuring the Granzyme B and TNF‐α expressions of T cells in the tumors of HFD‐fed mice treated with coixenolide or vehicle control. *n* = 4 mice per group. Data are mean ± SD; a two‐sided *t*‐test was used. All *p*‐values are two‐sided. ns, not significant; *p < 0.05.

Next, we compared the grade of infiltration for each cell subtype in distinct samples; cytotoxic CD8^+^ T, Treg, and Tfh cells were the most abundant (Figure 3D; Figure S2A,B). Coixenolide specifically and significantly augmented the infiltration of cytotoxic CD8^+^ T cells in tumors of the HFD group (Figure 3E), consistent with the findings of the RNA‐seq analysis (Figure 1). Immunofluorescence staining showed a similar result, with a large number of CD8^+^ T cells emerging in tumors of HFD‐fed but not CD‐fed mice after coixenolide treatment (Figure 3F). Furthermore, coixenolide attenuated the infiltration of Tregs in tumors of the HFD‐fed mice, which might contribute to augmenting anti‐PAAD immunity (Figure S2C).

To ascertain whether coixenolide enhanced the function of tumor‐infiltrating cytotoxic CD8^+^ T cells besides promoting their infiltration, we assessed the expression levels of genes related to T cell activation (*Cd8a* and *Cd69*), cytotoxic cytokines (*Ifng*, *Prf1*, and *Gzmb*), and immune checkpoint receptor (*Pdcd1*) following coixenolide treatment, which confirmed the coixenolide‐enhanced activation and cytotoxicity (Figure 3G). However, in the CD‐fed mice, coixenolide did not significantly alter the infiltration of cytotoxic CD8^+^ T cells and had no significant impact on their functionality either (Figure 3H). Next, we further extracted the cytotoxic CD8^+^ T cells subset and conducted a comparative analysis of gene expression alterations following coixenolide administration (Figure S2D). GO enrichment analysis of the DEGs was performed to determine the biological features. Notably, the upregulated genes in cytotoxic CD8^+^ T cells in the HFD group were significantly enriched in GO terms associated with T cell proliferation and activation (Figure S2E). Subsequently, we reordered the CD8^+^ T cells into pseudo‐time trajectories using Monocle2. Although a distinct subset of naive CD8^+^ T cells was not delineated, coixenolide significantly suppressed the exhaustion of cytotoxic CD8^+^ T cells (Figure 3I). Consistently, after coixenolide treatment, tumor‐infiltrating CD8^+^ T cells had significantly higher TNF‐α and GZMB production in HFD‐fed mice, indicating that coixenolide increased the cytotoxic cytokine production of CD8^+^ T cells and enhanced their antitumor activity in vivo (Figure 3J; Figure S2F).

### 2.4 Coixenolide Inhibited S100a8/a9^+^ Cancer Cells in HFD‐Fed Mice

Since pancreatic cancer cells originate from epithelial cells, epithelial cells were isolated to establish a novel gene‐cell matrix. We conducted separate clustering analysis and identified 21 original clusters based on our scRNA‐seq data (Figure 4A). To further identify malignant epithelial cells in PAAD, we inferred scCancer and calculated malignant score for each epithelial cluster [28]. T cells were used as reference cells, which had no malignant score in PAAD. All epithelial clusters exhibited significantly elevated malignant scores compared to reference cells, indicating that all the epithelial cells derived from the tumor were malignant cells (Figure 4B; Figure S3A). We also conducted a comparative analysis of the proportions of individual malignant epithelial cell clusters in tumors between the HFD and CD groups, which revealed a significant inter‐sample heterogeneity of tumor cells in PAAD (Figure 4C).

**FIGURE 4:**
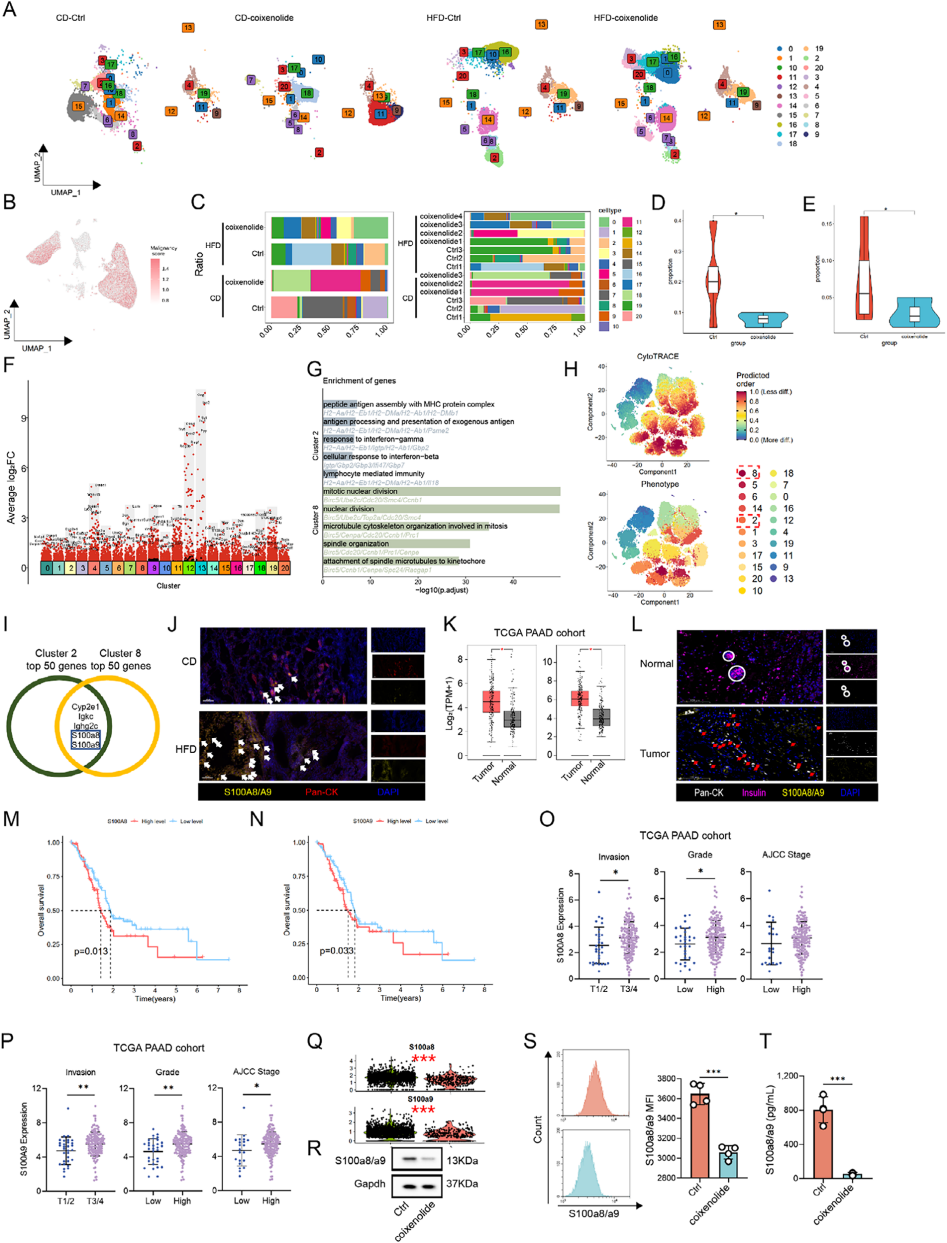
Transcriptomic analyses revealed that coixenolide significantly inhibited *S100a8/a9* ^+^ PAAD cells in HFD‐fed mice. (A) Subclusters of epithelial cells. (B) Malignancy score of epithelial cells. (C) The heterogeneity of epithelial cell clusters among different groups and tissue samples based on cell ratio. (D,E) Changes in the proportions of Cluster 2 (D) and Cluster 8 (E) tumor cells. *n* = 7 for HFD group and *n* = 6 for CD group. Data are presented as mean ± SD; a two‐sided *t*‐test was used. (F) The upregulated genes across 21 tumor cell subsets. (G) Pathway enrichment associated with the top 100 upregulated genes (ranked by *p*‐value) of Clusters 2 and 8 tumor cells. (H) CytoTRACE score and distribution of tumor cell clusters. (I) Shared genes of Clusters 2 and 8. (J) The colocalization of S100A8/A9 (yellow), Pan‐CK (red), and DAPI (deep blue) in the tumor tissues of CD‐ and HFD‐fed mice; representative of three independent experiments. (K) The expression patterns of S100A8 and S100A9 between tumor and normal pancreatic tissues using the GEPIA webtool; *n* = 179 (tumor tissue) and 171 (normal tissue); a two‐sided Wilcoxon test was used. (L) The colocalization of S100A8/A9 (yellow), insulin (pink), Pan‐CK (white), and DAPI (deep blue) in the peritumoral pancreatic tissue, as well as in tumor tissues in PAAD patients. (M,N) Kaplan–Meier survival plot indicating that patients with high levels of S100A8 (M) or S100A9 (N) have a worse clinical outcome in the TCGA PAAD cohort; log‐rank test was used. (O,P) The expressions of S100A8 (O) and S100A9 (P) between patients with PAAD of different invasion levels, grades, and AJCC stages. The data were sourced from the TCGA cohort; a two‐sided *t*‐test was used. (Q) Expression levels of *S100a8* and *S100a9* in Clusters 2 and 8 tumors of HFD‐fed mice following coixenolide treatment. A two‐sided *t*‐test was used. (R,S) Western blot (R) and flow cytometry plot (S) measuring *S100a8/a9* expression in Pan02 cells following coixenolide treatment. *n* = 4 independent experiments; two‐sided *t*‐test was used. (T) The levels of *S100a8/a9* in the supernatants of Pan02 cells with or without coixenolide treatment were determined using ELISA. *n* = 3 independent experiments; two‐sided *t*‐test was used. *p < 0.05, **p < 0.01, ***p < 0.001.

To investigate the effect of coixenolide on tumor cell populations, we compared the changes of the 21 malignant epithelial cell clusters in tumors from the HFD and CD groups with and without coixenolide treatment. Remarkably, coixenolide prominently attenuated the presence of clusters 2 and 8 within tumors of HFD‐fed mice (Figure 4D,E). To further elucidate the biological characteristics of clusters 2 and 8, we performed GO enrichment analysis of their top 100 highly expressed genes (Figure 4F). There was a significant enrichment of highly expressed genes with GO terms related to “MHC protein complex” and “response to immune cytokines” in cluster 2, suggesting its potential involvement in interactions with immune cells. Cluster 8 had significant enrichment for characteristics related to “nuclear division” (Figure 4G). Subsequently, we employed CytoTRACE to investigate the developmental potential of the malignant epithelial clusters. Notably, these two clusters exhibited the highest CytoTRACE score, indicating a significant stemness potential (Figure 4H; Figure S3B). Therefore, we speculated that these two clusters were crucial for pancreatic tumorigenesis in HFD‐fed mice.

To explore the shared characteristics of the tumor clusters 2 and 8, we performed an intersection analysis using the top 50 upregulated DEGs in both clusters to identify common hub genes. Both clusters 2 and 8 exhibited elevated expression of *S100a8* and *S100a9* compared to other clusters (Figure 4I; Figure S3C). S100A8 and S100A9 always bind to each other and change their self‐structure to form polymers (hereafter referred to collectively as S100A8/A9) [29]. Remarkably, these two tumor clusters displayed significantly higher abundance in tumors in the HFD group compared to the CD group (Figure 4A). Multiplex immunofluorescence staining further confirmed a significant enrichment of *S100a8/a9^+^* cancer cells in the TME in the HFD group compared to the CD group (Figure 4J). Similarly, we observed that exposure to adipocyte‐conditioned medium for 48 h significantly upregulated *S100a8/a9* expression in Pan02 cells (Figure S3D). To investigate the differential expression of S100A8/A9 in patients with PAAD, we further analyzed The Cancer Genome Atlas (TCGA) data using the online tool Gene Expression Profiling Interactive Analysis (GEPIA). There was significantly higher expression of S100A8/A9 at tumor sites compared with para‐cancerous sites in PAAD (Figure 4K). This was confirmed by immunofluorescence staining of patient samples, showing the enhanced accumulation of S100A8/A9^+^ cells in tumor tissues compared to peritumoral pancreatic tissues (Figure 4L). Patients with high S100A8/A9 expression in tumor had significantly poorer survival (Figure 4M,N) [30]. Moreover, S100A8/A9 expression was higher in advanced‐stage tumors than early‐stage tumors, and positively associated with histologic grade and invasion depth (Figure 4O,P).

To further evaluate S100A8/A9 expression and its association with the clinical outcomes of patients with PAAD, we analyzed a published scRNA‐seq dataset from the Genome Sequence Archive (GSA) database including 24 pancreatic cancer samples and 11 controls without any treatment [31]. The cells were classified into 10 major types according to the labels from the dataset (Figure S3E). We compared the expression level of S100A8/A9 across cell types, and high S100A8/A9 expression was observed in macrophages as expected. S100A8/A9 was also highly expressed in type 2 ductal cells (Figure S3F). Considering type 1 ductal cells as normal and type 2 as malignant [31], we conducted a comparative analysis of S100A8/A9 expression between these two cell types. Compared with type 1 cells, malignant type 2 cells exhibited a significantly higher expression of S100A8/A9 (Figure S3G).

Our scRNA‐seq results demonstrated that the expression levels of *S100a8/a9* were significantly decreased in Clusters 2 and 8 following coixenolide administration, which was further validated by qPCR analysis (Figure 4Q; Figure S3H). Transcriptional analysis of scRNA‐seq data further revealed that coixenolide downregulated the transcriptional activity of *S100a8/a9*, with enriched pathways related to transcriptional regulation significantly altered (Figure S3I). Accordingly, coixenolide inhibited the protein expression and surface level of *S100a8/a9* in tumor cells (Figure 4R,S). As S100A8/A9 not only exists in cancer cells but also can be secreted into the extracellular microenvironment, we assessed the protein level of *S100a8/a9* in the culture media; the secretion of *S100a8/a9* was similarly suppressed following coixenolide treatment (Figure 4T). Additionally, analysis of scRNA‐seq‐derived transcriptional signatures indicated that coixenolide‐modulated genes were involved in post‐translational modification pathways, with particularly enhanced ubiquitination‐related gene expression (Figure S3J).

We further examined the protein expression of the three other genes (*Igkc*, *Cyp2e1*, and *Ighg2c*) that were also found to be highly expressed in both Cluster 2 and Cluster 8 (Figure 4I). Western blot analysis revealed that the expression levels of these proteins remained unchanged after coixenolide treatment (Figure S3K).

### 2.5 Coixenolide Enhanced CD8^+^ T Cell Function by Intervening in the Interaction Between S100a8/a9^+^ Cancer Cells and CD8^+^ T Cells

There are complex cell‐to‐cell communications among various cell subpopulations in the TME that together facilitate tumor progression. To clarify the cell–cell interaction mechanisms, we built separate intercellular communication networks between tumor‐infiltrating T cells and malignant epithelial cells in the TME of tumors in the HFD‐fed mice with or without coixenolide treatment (Figure 5A). Malignant cells in clusters 2 and 8 had the highest incoming and outgoing interaction strengths (Figure 5B), indicating their active biological properties. Outgoing communication pattern analysis also suggested coordinated signaling between clusters 2 and 8 (Figure 5C). Notably, among all investigated cell types, cells within Clusters 2 and 8, which highly expressed *S100a8/a9* displayed the strongest outgoing signaling capacity toward CD8^+^ T cells (Figure 5B; Figure S4A–C). Furthermore, we quantified the absolute number of *S100a8/a9* ^+^ cells within tumor cells, monocytes, macrophages, and neutrophils. Tumor cells harbored the largest proportion of *S100a8/a9^+^* cells (Figure S4D). Therefore, we hypothesized a strong interaction between cytotoxic CD8^+^ T cells and cancer cells in clusters 2 and 8.

**FIGURE 5:**
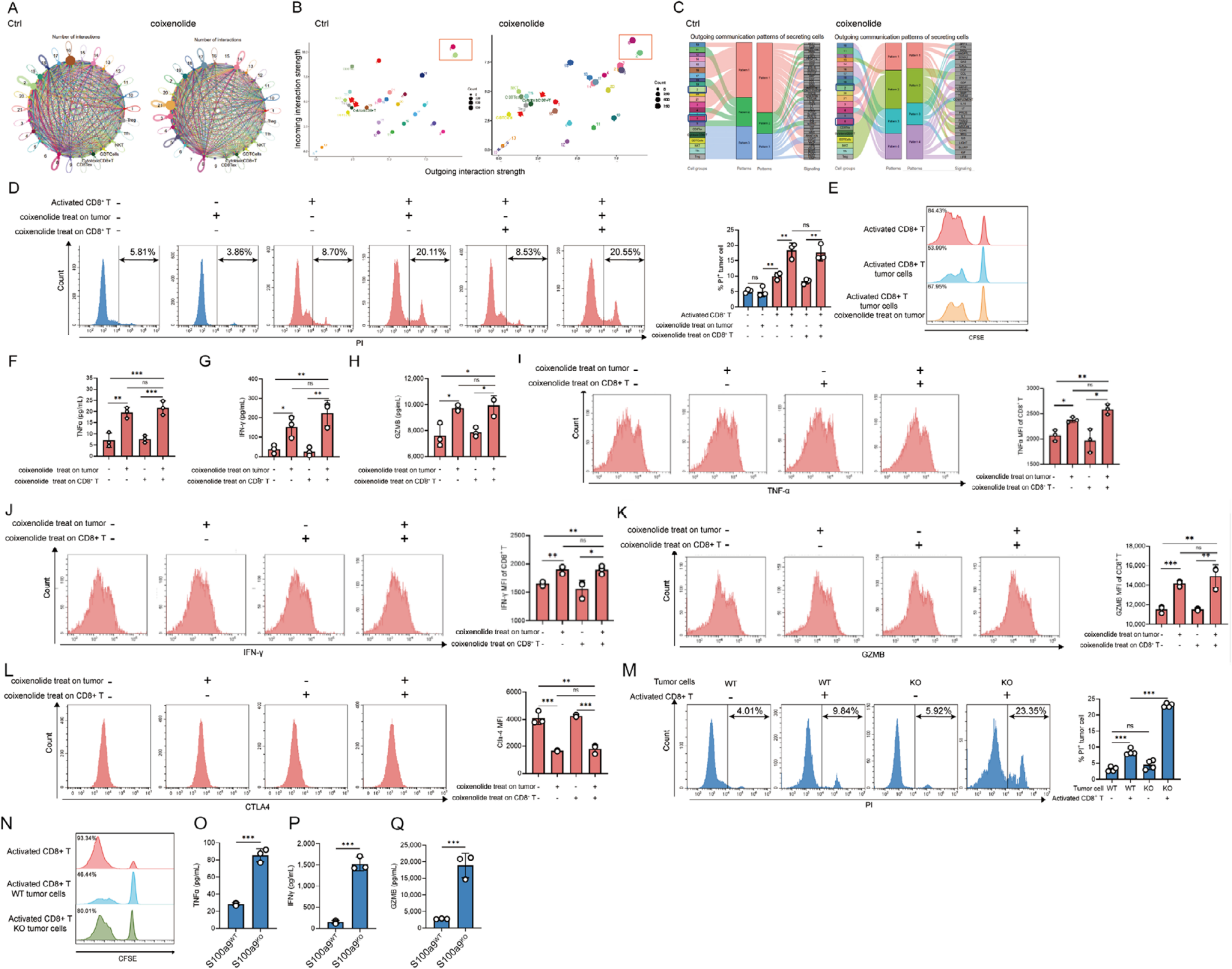
Cell–cell communication analysis indicated that *S100a8/a9* served as a critical target for coixenolide, regulating the interactions between PAAD cells and CD8^+^ T cells. (A) The overview of cell–cell interactions between malignant epithelial cells and T cells in the tumor tissues treated with vehicle control and coixenolide, respectively. (B) The intensity of intercellular signaling, showing both the sent and received signals. The cells in the upper right corner exhibit the strongest incoming and outgoing interactions. *S100a8/a9* ^+^ tumor cell clusters (Clusters 2 and 8) are highlighted in red boxes. Cytotoxic CD8^+^ T and Treg cells are indicated by red arrows. (C) The outgoing communication patterns of epithelial cells and T cells in the tumor of HFD‐fed mice with or without coixenolide treatment. (D) CD8^+^ T cell killing assays were conducted using coculture of Pan02 and CD8^+^ T cells. Pan02 or CD8^+^ T cells were pretreated with 25 mg/mL coixenolide before coculture. (E) Primary CD8^+^ T cells were activated by incubation with CD3/CD28 antibody and labeled with carboxy‐fluorescein succinimidyl ester (CFSE). (F–H) The levels of TNF‐α (F), IFN‐γ (G), and Granzyme B (GZMB) (H) in the supernatants after co‐culture of CD8^+^ T and Pan02 cells pretreated with coixenolide (+) or vehicle control (−) were determined using ELISA. (I–K) The expression of TNF‐α (I), IFN‐γ (J), and Granzyme B (GZMB) (K) in CD8^+^ T cells after co‐culture of CD8^+^ T cells and Pan02 cells pretreated with coixenolide (+) or vehicle control (−) was determined using flow cytometry. (L) Flow cytometry analyses of T cell exhaustion markers (CTLA‐4) on CD8^+^ T cells after coculture of Pan02 and CD8^+^ T cells pretreated with coixenolide (+) or vehicle control (−). (M,N) Flow cytometry analyses of CD8^+^ T cell killing (M) and division (N) activities. CD8^+^ T cells were cocultured with *S100a9* knockout (KO) or wild‐type (WT) Pan02 cells. (O–Q) The levels of TNF‐α (O), IFN‐γ (P), and GZMB (Q) in the supernatants after coculture of CD8^+^ T cells with WT or *S100a9* KO Pan02 cells were determined using ELISA. *n* = 3 or 4 independent coculture experiments or replicates. One‐way ANOVA with Tukey's post‐hoc test (E–L), two‐sided *t*‐test (M–N), or two‐way ANOVA (O‐Q) was used. All *p* values are two‐sided. *p < 0.05, **p < 0.01, ***p < 0.001.

To identify the impact of coixenolide on CD8^+^ T cell cytotoxicity, we designed an in vitro co‐culture assay. We first purified CD8^+^ T cells from the spleen and pre‐stimulated them with immobilized anti‐CD3/CD28 and IL‐2 cytokine. We then exposed CD8^+^ T or Pan02 pancreatic cancer cells to coixenolide. Subsequently, we established a co‐culture system of CD8^+^ T and cancer cells after coixenolide treatment. We first showed low cytotoxicity of coixenolide on both CD8^+^ T and tumor cells (Figure S4E,F). In the co‐culture of CD8^+^ T and tumor cells with coixenolide pretreatment of either cell separately, coixenolide enhanced the proliferation and tumor‐killing effects of CD8^+^ T cells through modulation of tumor cells, but not directly of CD8^+^ T cells; coixenolide treatment of only CD8^+^ T cells did not have a notable effect on their activity or cytotoxicity (Figure 5D–L). Consistently, in vitro CD8^+^ T cell neutralization abolished the activation of CD8^+^ T cells and the antitumor effect enhanced by coixenolide (Figure S4G,H). Coixenolide administration significantly reduced the infiltration of Treg cells in tumor tissues (Figure S2C). To further explore the effect of coixenolide on Treg cells and its subsequent impact on CD8^+^ T cells, we treated Treg cells with coixenolide in vitro for 24 h. Coixenolide treatment led to a significant decrease in the functional markers of Treg cells (Figure S4I). Furthermore, in the co‐culture of Treg and CD8^+^ T cells, only a partial enhancement in CD8^+^ T cell function, with a significant change in the production of IFN‐γ but not TNF‐α or GZMB, was observed in the group where Treg cells were pretreated with coixenolide, as compared with the control group (Figure S4J). Collectively, our results suggested that coixenolide exerted a certain regulatory effect on Treg cells. Nevertheless, disrupting the immunosuppressive crosstalk targeting CD8^+^ T cells may represent a significant mechanism by which coixenolide enhances cytotoxic CD8^+^ T cell function.

Our previous analyses suggested that coixenolide inhibited S100A8/A9^+^ tumor cells, which was strongly associated with interactions with cytotoxic T cells. We therefore sought to determine whether inhibition of S100A8/A9 in tumor cells could activate cytotoxic T cells. We generated Pan02 and CD8^+^ T cell co‐culture models and administered the *S100a9* inhibitor tasquinimod to the tumor cells, resulting in a significant enhancement of CD8^+^ T cell activity and cytotoxicity (Figure S5A–D). To further interrogate the specific functions of S100A8/A9, we knocked out (KO) *S100a9* in wild‐type (WT) Pan02 cells using CRISPR‐Cas9‐mediated gene knockout with a specific single‐guide RNA (*sgS100a9*) [32], with a reduction in *S100a8/a9* confirmed at the protein level (Figure S5E). Knockout of *S100a9* in tumor cells did not directly affect cell viability (Figure S5F). The CD8^+^ T cells activated *ex vivo* were cocultured with WT and *S100a9* KO tumor cells, respectively. The activity and cytotoxicity of CD8^+^ T cells were significantly enhanced when cocultured with *S100a9* KO tumor cells compared to with WT cells (Figure 5M–Q).

### 2.6 Coixenolide Enhanced CD8^+^ T Cell Infiltration and Cytotoxicity in a S100A8/A9‐CD36 Dependent Manner

When secreted, S100 proteins interact with several receptors, including the fatty acid transporter CD36 [33], Toll‐like receptor 4 (TLR4) [34], and the receptor for advanced glycation end products (RAGE) [35]. To elucidate the mechanism underlying the inhibition of the activity and cytotoxicity of CD8^+^ T cells mediated by tumor cells‐derived S100A8/A9, we analyzed our scRNA‐seq data on the changes in the expression of the *S100a8/a9* receptor on CD8^+^ T cells following coixenolide treatment; *Cd36* was significantly inhibited in CD8^+^ T cells following coixenolide treatment, which suppressed S100A8/A9 of cancer cells (Figure 6A). In the TCGA PAAD cohort, the expression of S100A8/A9 was significantly positively associated with the expression of CD36 (Figure 6B). Immunofluorescence staining of patient samples confirmed this observation, demonstrating a significantly elevated expression level of CD36 in CD8^+^ T cells surrounding S100A8/A9^+^ tumor cells in cancerous tissues (Figure 6C). Preconditioning tumor cells with coixenolide reduced the surface protein level of *Cd36* on CD8^+^ T cells and impaired the binding between *S100a8/a9* and *Cd36* on CD8^+^ T cells (Figure 6D,E).

**FIGURE 6:**
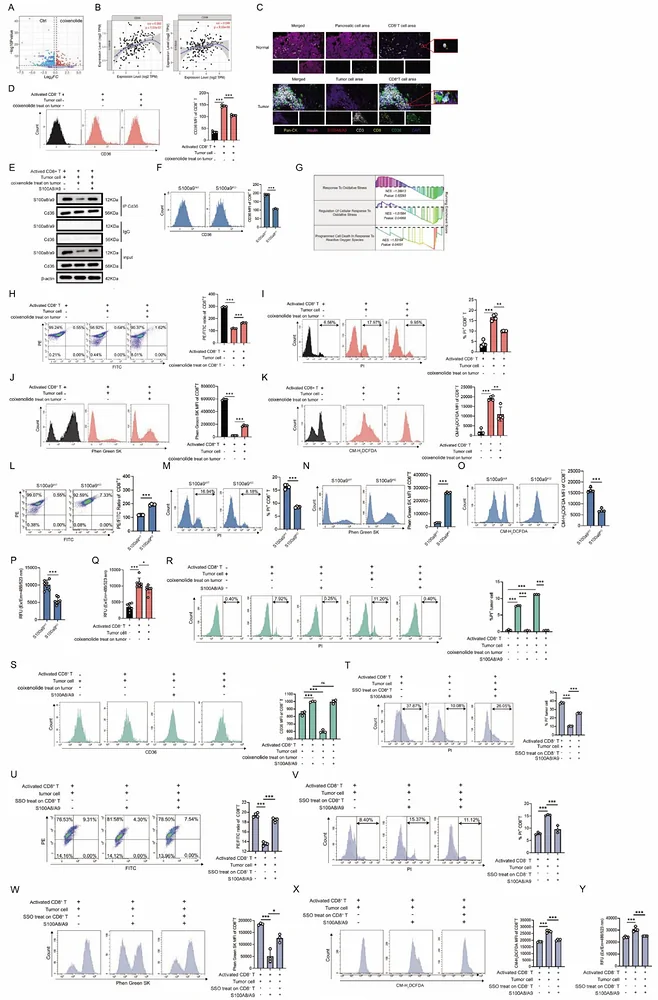
Coixenolide regulated the transcriptional and metabolic programs (including lipid peroxidation) of tumor‐infiltrating CD8^+^ T cells through modulating the S100A8/A9‐CD36 signaling axis. (A) The expression of *S100a8/a9* receptors in tumor‐infiltrating CD8^+^ T cells from tumors of HFD‐fed mice following coixenolide administration. (B) Co‐expression of S100A8 and S100A9 with CD36 in the TCGA PAAD cohort. (C) Immunofluorescent staining showing colocalization of S100A8/A9 (red), insulin (pink), CD3 (white), CD8 (yellow), CD36 (green), and DAPI (deep blue) in peritumoral pancreatic tissues (lower), as well as Pan‐CK (white) in pancreatic tumor tissues in PAAD patients. (D) Flow cytometry of *Cd36* expression in CD8^+^ T cells cocultured with Pan02 cells. (E) Co‐immunoprecipitation analysis of *Cd36*‐*S100a8/a9* interaction in cocultures of activated CD8^+^ T cells and tumor cells with or without coixenolide treatment. (F) Flow cytometry of *Cd36* expression on CD8^+^ T cells cocultured with *S100a9* KO/WT Pan02 cells. (G) Single‐cell sequencing analysis of tumor‐infiltrating CD8^+^ T cells in HFD‐fed mice. (H–K) Flow cytometry analyses of lipid peroxidation (H), cell death (I), iron (J), and cytosolic ROS (K) in CD8^+^ T cells cocultured with Pan02 cells. (L–O) Lipid peroxidation (L), cell death (M), iron (N), and cytosolic ROS (O) in CD8^+^ T cells cocultured with *S100a9* KO/WT Pan02 cells. (P,Q) Fatty acid uptake assay on CD8^+^ T cells cocultured with WT or *S100a9* KO Pan02 cells (P) and CD8^+^ T cells cocultured with Pan02 cells pretreated with coixenolide (+) or vehicle control (−) (Q). (R,S) Flow cytometry analyses of cell death (R) and *Cd36* expression (S) in CD8^+^ T cells cocultured with Pan02 cells pretreated with coixenolide or S100A8/A9 recombinant protein. (T–Y) Flow cytometry analyses of killing activity (T), lipid peroxidation (U), cell death (V), iron (W), cytosolic ROS (X), and free fatty acid (Y) in CD8^+^ T cells. CD8^+^ T cells were pretreated with 50 µm CD36 inhibitor SSO (+) or DMSO (−) for 24 h before coculture with Pan02 cells. Additionally, coculture models were treated with S100A8/A9 recombinant protein (+) or DMSO (−). *n* = 3 or 4 independent coculture experiments or replicates. One‐way ANOVA with Tukey's post‐hoc test, two‐sided *t*‐test, or two‐way ANOVA was used where appropriate. All *p*‐values are two‐sided. *p < 0.05, **p < 0.01, ***p < 0.001.

Consistently, *S100a9* KO led to a significant reduction in *Cd36* expression on CD8^+^ T cells (Figure 6F). We further performed multiplex immunofluorescence (mIF) staining to examine *S100a8/a9* expression in macrophages and neutrophils. Coixenolide treatment did not significantly alter *S100a8/a9* levels in these myeloid populations in the tumor tissues in both the CD and HFD groups. Moreover, no obvious colocalization was observed between *S100a8/a9*‐positive macrophages/neutrophils and *Cd36*‐positive CD8^+^ T cells (Figure S6A,B). To further investigate whether the S100A8/A9‐TLR4/RAGE signaling axis mediates the effects of coixenolide, we first examined the expression levels of TLR4 and RAGE on CD8^+^ T cells following coixenolide treatment. The expression of TLR4 and RAGE on CD8^+^ T cells was elevated following co‐culture with tumor cells, while no significant changes in TLR4 or RAGE expression were detected after coixenolide treatment (Figure S6C,D). Notably, treatment with an agonist of TLR4 (LPS) or RAGE (AGEs) alone did not significantly impact the function of CD8^+^ T cells when co‐cultured with PAAD cells. Moreover, the addition of LPS or AGEs did not further modify the enhancement of CD8^+^ T cell function induced by coixenolide (Figure S6E,F). These indicate that under the co‐culture conditions of tumor and CD8^+^ T cells, activation of TLR4 or RAGE signaling is insufficient to have a significant influence on the function of CD8^+^ T cells when exposed or not exposed to coixenolide.

S100A8/A9 could bind to CD36 and promote the transport and uptake of fatty acids [36, 37]. In the TME, fatty acids induce CD8^+^ T cell ferroptosis in a CD36‐dependent manner [38]. We further characterized lipid and fatty acid uptake, lipid peroxidation, and ferroptosis‐related changes in cytotoxic CD8^+^ T cells of HFD‐fed mice following coixenolide treatment based on scRNA‐seq data. In the coixenolide‐treated group, cytotoxic CD8^+^ T cells exhibited gene expression patterns associated with reduced oxidative stress response and decreased cell death in response to reactive oxygen species (ROS) compared to the control group (Figure 6G). Moreover, gene expression analysis further demonstrated that cytotoxic CD8^+^ T cells in the coixenolide group had lower expression of genes associated with ferroptosis activation, and meanwhile had higher expression of genes associated with ferroptosis inhibition compared with the control group (Figure S6G,H).

We also analyzed the scRNA‐seq data of tumor‐infiltrating CD8^+^ T cells in patients with PAAD to confirm the clinical relevance of our findings. Patients with PAAD were divided into two distinct cohorts based on S100A8/A9 expression levels in tumor cells (high‐ and low‐S100A8/A9 expression groups) (Figure S6I,J). To investigate the CD8^+^ T cell function in these two patient groups, we further defined CD8^+^ T cell subgroups according to the corresponding cellular markers (Figure S6K). Consistent with findings in mice, tumor‐infiltrating CD8^+^ T cells from patients with high tumor S100A8/A9 expression exhibited enhanced activation of signaling pathways involved in ROS, iron ion, and oxidative stress responses, as well as lipid transport and lipid responses, compared to patients with low S100A8/A9 expression (Figure S6L,M). Gene expression analysis showed that genes associated with lipid peroxidation or ferroptosis activation were enriched in tumor‐infiltrating CD8^+^ T cells in S100A8/A9^high^ patients compared to S100A8/A9^low^ patients (Figure S6N). Moreover, tumor‐infiltrating CD8^+^ T cells in S100A8/A9^high^ patients had lower expression of genes associated with cytotoxicity of T cells and higher expression of genes associated with exhaustion (e.g., CTLA‐4, PD‐1, and LAG3) (Figure S6O,P).

We then used a lipid peroxidation assay kit and cell death ratio to evaluate the level of ferroptosis in CD8^+^ T cells. The sensor in the lipid peroxidation kit changes its fluorescence from R‐Phycoerythrin (PE) to fluorescein isothiocyanate (FITC) upon peroxidation by lipid ROS in cells, thus enabling ratio‐metric measurement of lipid peroxidation. As the PE‐to‐FITC ratio drops, the level of lipid peroxidation increases. CD8^+^ T cells cocultured with tumor cells pretreated with coixenolide for 24 h exhibited diminished levels of lipid peroxidation (Figure 6H), reduced ratios of cell death (Figure 6I), decreased levels of iron suggested by higher fluorescence intensity (Figure 6J), and lower cytosolic ROS levels (Figure 6K). We observed similar trends in CD8^+^ T cells cocultured with *S100a9* KO tumor cells (Figure 6L–O). We also used a fatty acids assay kit to detect fatty acid uptake in the cells. CD8^+^ T cells cocultured with *S100a9* KO tumor cells had less free fatty acid uptake compared to those cocultured with WT cells (Figure 6P). A similar pattern was identified in CD8^+^ T cells cocultured with coixenolide‐pretreated tumor cells (Figure 6Q). These findings suggested that inhibiting S100A8/A9 in tumor cells could reverse CD36‐mediated lipid peroxidation and ferroptosis in tumor‐infiltrating CD8^+^ T cells.

To further confirm whether coixenolide activated CD8^+^ T cells through the S100A8/A9‐CD36 axis, we supplemented the coculture model with additional recombinant S100A8/A9 proteins. The addition of recombinant S100A8/A9 significantly inhibited the cytotoxicity of CD8^+^ T cells and upregulated *Cd36* expression of CD8^+^ T cells in the coculture model following pretreatment of tumor cells with coixenolide (Figure 6Q,S). Further treatment of CD8^+^ T cells with the CD36 inhibitor sulfosuccinimidyl Oleate sodium (SSO) significantly reversed the functional impairment of CD8^+^ T cells (Figure 6T), as well as lipid peroxidation, cell death, ferroptosis, and free fatty acid uptake induced by the additional supplementation of S100A8/A9 (Figure 6U–Y). We also established *Cd36*‐knockout (KO) CD8^+^ T cells, which showed markedly attenuated free fatty acid uptake, lipid peroxidation, cell death, and ferroptosis when compared to wild‐type (WT) CD8^+^ T cells (Figure S6R–V). Coixenolide treatment did not further induce significant changes in these aspects of *Cd36‐*KO CD8^+^ T cells [39]. Furthermore, we established a fatty acid‑deprived coculture system by replacing FBS with Insulin‐Transferrin‐Selenium (ITS‐G, 100×). Under fatty acid‑free conditions, tumor cells barely induced lipid peroxidation and ferroptosis of CD8^+^ T cells (Figure S7A–E), and exogenous S100A8/A9 protein, SSO pretreatment of CD8^+^ T cells, or coixenolide treatment of tumor cells no longer significantly altered the levels of lipid peroxidation and ferroptosis of CD8^+^ T cells (Figure S7F–J).

These findings suggested that coixenolide reversed the increased levels of fatty acid uptake and ferroptosis of CD8^+^ T cells induced by tumor cells by inhibiting the S100A8/A9‐CD36 signaling axis.

### 2.7 S100a9 Knockout Significantly Activated Cytotoxic T Cells and Suppressed PAAD In Vivo

To further assess the impact of coixenolide on S100A8/A9 expression in tumor cells and its role in reversing CD36‐mediated lipid peroxidation and ferroptosis of CD8^+^ T cells, we conducted in vivo experiments by orthotopically transplanting *S100a9* WT and KO (using the CRISPR‐Cas9 technique) Pan02 cells into the pancreas of immunocompetent HFD‐ or CD‐fed C57BL/6 mice, followed by administration of either saline or coixenolide (Figure 7A). Consistent with the in vitro findings, C57BL/6 mice bearing *S100a9* KO Pan02 tumors had decreased tumor volume and tumor weight (Figure 7B–G). Notably, for tumors of HFD‐fed mice, while knocking out *S100a9* exhibited a significantly enhanced tumor‐suppressive effect, coixenolide did not further suppress the *S100a9* KO tumor growth (Figure 7B–D). However, in CD‐fed mice, coixenolide further inhibited the growth of *S100a9* KO tumors, indicating that coixenolide might exert additional inhibitory effects on tumors in CD‐fed mice through other pathways (Figure 7E–G). Flow cytometry revealed that the proportion of intra‐tumoral CD8^+^ T cells was increased in HFD‐fed mice with (coixenolide‐treated) *S100a9* KO PAAD compared to those with a WT tumor (Figure 7H,I). Although less pronounced than in HFD‐fed mice, knockout of *S100a9* (combined with coixenolide treatment) resulted in a modest increase in the proportion of CD8^+^ T cells within tumors in CD‐fed mice (Figure S8A,B). Furthermore, compared to WT tumors, CD8^+^ T cells within *S100a9* KO tumors with or without coixenolide treatment produced more anticancer cytotoxic effectors, including IL‐2, GZMB, perforin, and TNF‐α (Figure 7J–K; Figure S8C,D). These findings further demonstrated that coixenolide‐mediated tumor suppression and improvement in the antitumor activity of CD8^+^ T cells in immunocompetent mice were due to inhibiting S100A8/A9 of PAAD cells.

**FIGURE 7:**
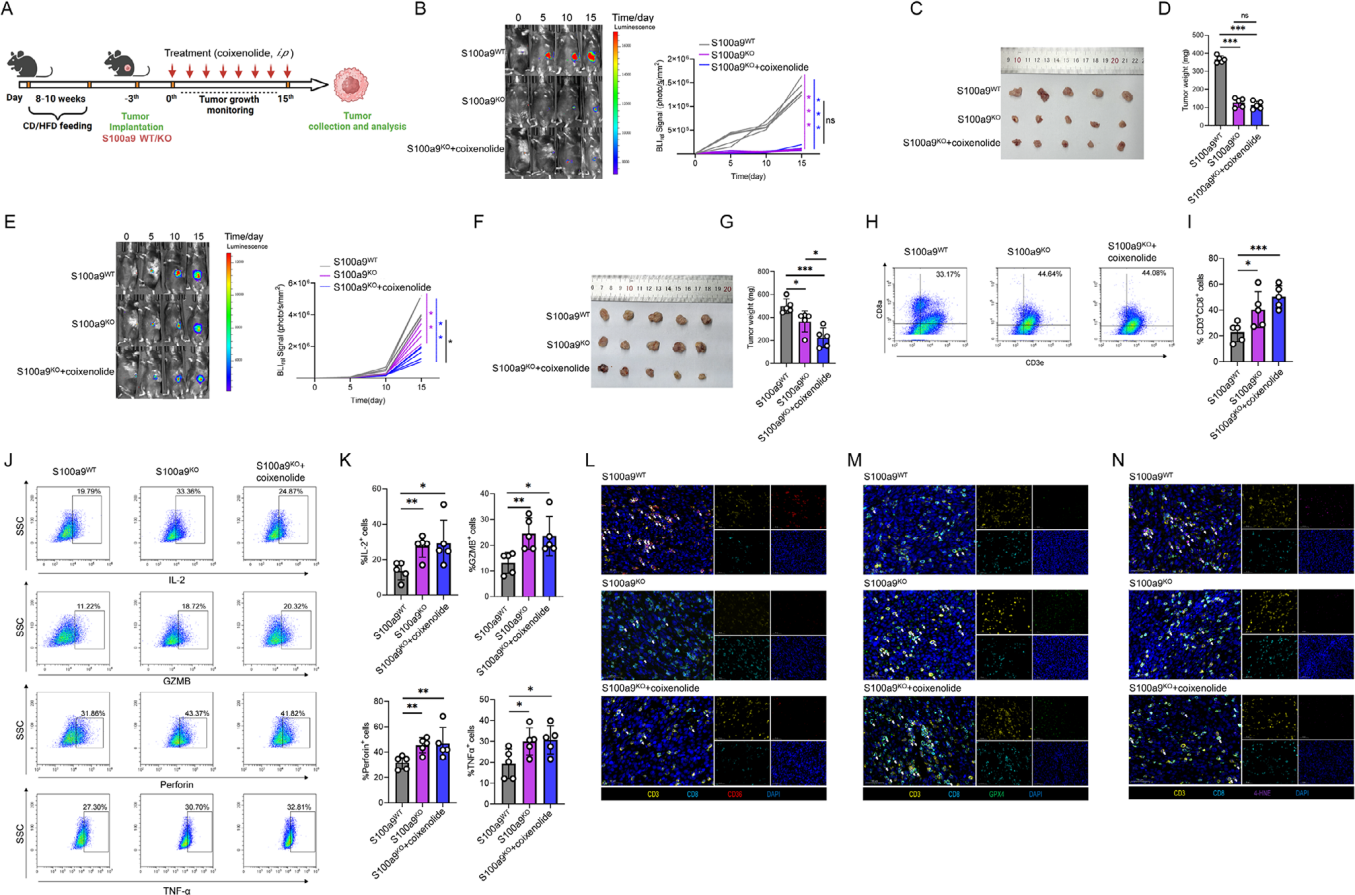
Knockout of *S100a9* markedly enhanced the activity of tumor‐infiltrating CD8^+^ T cells. (A) Schematic diagram depicting the experimental setup. (B–D) In vivo imaging (B), tumor picture (C), and tumor weight (D) of *S100a9* WT or KO pancreatic tumors with or without 2.5 g/kg coixenolide treatment in HFD‐fed mice. *n* = 5 mice per group. Data are presented as mean ± SD; two‐way ANOVA with Tukey's post‐hoc was used. (E–G) In vivo imaging (E), tumor picture (F), and tumor weight (G) of *S100a9* WT or KO pancreatic tumors with 2.5 g/kg coixenolide or vehicle control treatment in CD‐fed mice. *n* = 5 mice per group. Data are presented as mean ± SD; two‐way ANOVA with Tukey's post‐hoc was used. (H) Flow cytometric analyses of CD8^+^ T cells in *S100a9* WT tumors and *S100a9* KO tumors with vehicle control or coixenolide treatment. (I) The quantification results of CD8^+^ T cell subpopulations isolated from *S100a9* WT tumors and *S100a9* KO tumors without and with coixenolide treatment. (J) Flow cytometric analyses of CD8^+^ T cell effector molecules including IL‐2, Granzyme B (GZMB), perforin, and TNF‐α in *S100a9* WT tumors and *S100a9* KO tumors without and with coixenolide treatment. (K) The quantification results of CD8^+^ T cell effector molecules isolated from *S100a9* WT tumors and *S100a9* KO tumors with coixenolide treatment. *n* = 5 mice per group; two‐sided *t*‐test was used. (L) Immunofluorescent staining showing colocalization of CD3 (yellow), CD8 (wathet), CD36 (red), and DAPI (deep blue) in *S100a9* WT tumors and *S100a9* KO tumors with coixenolide treatment. Scale bars, 50 µm. (M) Immunofluorescent staining showing colocalization of CD3 (yellow), CD8 (wathet), GPX4 (green), and DAPI (deep blue) in *S100a9* WT tumors and *S100a9* KO tumors with coixenolide treatment. Scale bars, 50 µm. (N) Immunofluorescent staining showing colocalization of CD3 (yellow), CD8 (white), 4‐HNE (pink), and DAPI (deep blue) in *S100a9* WT tumors and *S100a9* KO tumors with coixenolide treatment. Representative of three independent experiments. Scale bars, 50 µm. *p < 0.05, **p < 0.01, ***p < 0.001. All tests are two‐sided.

Our in vitro data suggested that coixenolide downregulated *Cd36* expression on CD8^+^ T cells through inhibition of tumor cell‐derived *S100a8/a9*, thereby mitigating lipid peroxidation and ferroptosis of CD8^+^ T cells. The in vivo models also confirmed that the *Cd36* expression of intra‐tumoral CD8^+^ T cells was decreased in *S100a9* KO PAADs compared to WT cancers (Figure 7L). To further support that *S100a9* knockout inhibited *Cd36* ‐ mediated lipid peroxidation and ferroptosis of intra‐tumoral CD8^+^ T cells, we labeled markers of ferroptosis (GPX4) and lipid peroxidation (4‐HNE) using multi‐immunofluorescence staining; the levels of ferroptosis and lipid peroxidation in CD8^+^ T cells of *S100a9* KO HFD‐fed mice with or without coixenolide treatment were significantly reduced (Figure 7M,N).

## 3 Discussion

Recent studies have found that dietary perturbations altering systemic metabolic state can impact anticancer immunity [40, 41]. Adaptation of tumor cells to HFD causes T cell dysfunction due to altered fatty acid partitioning and local depletion of essential metabolites [17, 42]. In the PyMT breast cancer model, obesity diminished CD8^+^ T cell function, which was associated with enhanced fatty acid oxidation (FAO) and weakened glycolysis in T cells [43]. Furthermore, impaired chemokine expression and amino acid metabolism associated with obesity inhibit the infiltration and effector function of CD8^+^ T cells [44]. Interestingly, a recent clinical study [42] demonstrated that obesity is associated with enhanced efficacy of PD‐1/PD‐L1 blockade against human cancer, a phenomenon referred to as the “obesity paradox”. This study demonstrated the significantly enhanced therapeutic efficacy of coixenolide against pancreatic cancer in HFD‐fed mice compared to CD‐fed mice, which was closely associated with the coixenolide‐induced activation of cytotoxic CD8^+^ T cells within the TME of HFD‐fed mice. Previous studies have demonstrated the antitumor and immunomodulatory properties of coixenolide [45]. However, the mechanism by which coixenolide activated CD8^+^ T cells in tumors of HFD‐fed mice was still unclear. Therefore, we further performed single‐cell sequencing. Cell–cell interaction analysis identified the ligand–receptor interaction between *S100a8/a9* of tumor cells and *Cd36* of CD8^+^ T cells as a crucial mechanism underlying coixenolide‐mediated activation of the immune response against PAAD in HFD‐fed mice. These were carefully validated using in vitro and in vivo studies.

S100A8 (also known as MRP‐8 or calgranulin‐A) and S100A9 (also known as MRP‐14 or calgranulin‐B) are members of the S100 protein family [46]. In pancreatic cancer, mutations and deletions of SMAD4 hinder stable expression of S100A8 [30, 31]. S100A8 interacts with S100A9, forming S100A8/A9, which is a secretory protein with elevated expression in PAAD and is significantly associated with patients' prognosis [47, 48, 49]. Previous studies [50, 51] suggested that in tumor tissues, S100A8/A9 was primarily secreted by myeloid‐derived suppressor cells (MDSCs), while fibroblasts and macrophages also exhibited elevated S100A8/A9 expression. In recent years, the expression of S100A8/A9 by tumor cells has been gradually elucidated [52]. Our scRNA‐seq data demonstrated the expression of *S100a8/a9* in pancreatic cancer cells, with a notable upregulation in HFD‐fed mice, a phenomenon likely driven by obesity‐associated factors released into the tumor microenvironment [53]. Clinical sequencing data similarly indicated a significant increase in S100A8/A9 expression in cancer cells. scRNA‐seq and in vitro data showed that coixenolide significantly inhibited *S100a8/a9* derived from tumor cells. Cell‐chat analysis revealed a significant interaction between high *S100a8/a9*‐expressing tumor cell populations and CD8^+^ T cells; however, little was known about its role in mediating the cytotoxic cytokine production and antitumor function of CD8^+^ T cells.

To further explore the effects of coixenolide on the interaction between tumor and CD8^+^ T cells, we initially analyzed the expression changes of S100A8/A9 receptors on CD8^+^ T cells using scRNA‐seq. TLR4 and RAGE are the major receptors that activate downstream signaling after interaction with S100A8/A9 [54]. Importantly, S100A8/A9 secreted by MDSCs could bind to the TLR4 receptor on the surface of CD8^+^ tumor‐infiltrating lymphocytes (TILs), inducing depletion and exhaustion of CD8^+^ TILs, an effect that could be reversed by anti‐TLR4 treatment [55]. However, we observed that coixenolide did not significantly alter TLR4 or RAGE expression in HFD‐fed mice, while it significantly inhibited *Cd36* expression of CD8^+^ T cells based on scRNA‐seq. Previous studies [33, 56] and data from the TCGA PAAD cohort demonstrated a strong correlation between CD36 and S100A8/A9 expression, and showed that S100A8/A9 could bind to CD36 to mediate lipid transport, thereby affecting lipid metabolism in various tumors. Consistently, our in vitro co‐culture model demonstrated that *S100a9* KO in tumor cells significantly downregulated the expression of *Cd36* on the surface of CD8^+^ T cells. Further investigation remains necessary to elucidate the impact of fatty acid uptake change mediated by S100A8/A9‐CD36 interaction on CD8^+^ T cell exhaustion in PAAD.

Metabolism is the driving force shaping T cell differentiation, function, and fate [57]. Emerging evidence [58] highlights the importance of maintaining metabolic fitness in securing the functional state of T cells. CD36 is a scavenger receptor functioning in lipid metabolism. In the immune system, CD36 mediates antigen acquisition and presentation by dendritic cells, and supports regulatory T cell function [59, 60]. Significantly, the uptake of fatty acids through CD36 could deplete the antitumor effector function of CD8^+^ T cells within the TME, through inducing ferroptosis, lipid peroxidation, and subsequent reduction in cytotoxic cytokine production by these cells [38]. Our data revealed that coixenolide treatment or *S100a9* knockout in tumor cells significantly mitigated ferroptosis of CD8^+^ T cells, thereby restoring their metabolic fitness and effector function. Importantly, the protective effects of coixenolide on CD8^+^ T cells were abrogated by exogenous supplementation of recombinant S100A8/A9, supporting that coixenolide acts directly through the S100A8/A9‐CD36 axis. This mechanistic link is particularly relevant in the context of a high‐fat diet (HFD), where exacerbated lipid accumulation in the tumor microenvironment (TME) hyperactivates this pathway, creating a state of metabolic vulnerability in CD8^+^ T cells [17, 61]. By targeting this axis, coixenolide not only reverses lipid peroxidation and ferroptosis but also unlocks a more robust antitumor immune response, especially in HFD‐induced tumors where the immunosuppressive lipid burden is most pronounced. Collectively, these findings support a novel interpretation that HFD enhances the S100A8/A9‐CD36‐induced immunosuppressive program in the TME, which can be targeted by coixenolide; this may explain the stronger therapeutic response observed in HFD‐fed mice.

Tumor‐infiltrating CD8^+^ T lymphocytes are key participants in cell‐mediated immune responses, and their clinical therapeutic potential has garnered significant attention [62]. However, PAAD exhibits a highly immunosuppressive TME [63]. Immunosuppressive cytokines secreted by tumor cells play a crucial role in evading immune recognition and cytotoxicity of CD8^+^ T cells, ultimately causing CD8^+^ T cell depletion [64]. Our research underscores the clinical application potential of coixenolide as an immunotherapeutic agent. Using single‐cell sequencing and both in vivo and in vitro models, we identified S100A8/A9 derived from cancer cells as a critical target of coixenolide in enhancing CD8^+^ T cell function. Additionally, we emphasize that targeting CD36‐mediated lipid peroxidation and ferroptosis represents a novel strategy to improve the anti‐PAAD efficacy of T cell‐based immunotherapy, and support CD36 as a promising immune checkpoint target. Furthermore, beyond the S100A8/A9‐CD36 axis identified in this study, using an anti‐CD25 mAb‐mediated Treg depletion model of HFD‐fed PAAD‐bearing mice, we imply that coixenolide may also regulate the crosstalk between Treg and CD8^+^ T cells, which may constitute an additional immunomodulatory route through which coixenolide reinforces antitumor immunity in the context of HFD‐associated PAAD (data not shown).

There are some limitations of this study. Relevant clinical translational investigations remain necessary to further validate our findings, particularly the therapeutic efficacy and synergistic effect of coixenolide in combination with immune checkpoint inhibitors (ICIs) already in clinical use. In this study, we confirmed through bioinformatics correlation analysis and in vitro co‐culture models that coixenolide administration significantly downregulated the expression of *Cd274* (encoding PD‐L1) of PAAD cells and *Pdcd1* (encoding PD‐1) of CD8^+^ T cells (Figure S9A,B). Furthermore, combination treatment with coixenolide and PD‐1 blockade (29F.1A12) markedly enhanced the cytotoxic and killing activities of CD8^+^ T cells against tumor cells (Figure S9C,D). The precise mechanism by which coixenolide inhibits tumor cell‐derived S100A8/A9 remains unclear. While our study establishes the S100A8/A9‐CD36 axis as a significant immune‐metabolic pathway through which coixenolide may enhance CD8^+^ T cell cytotoxicity, future investigation focusing on delineating how coixenolide regulates Treg cells within the pancreatic tumor microenvironment may further clarify the underlying mechanism.

## 4 Conclusions

This study demonstrated for the first time that coixenolide might suppress obesity‐associated PAAD via activating the function of intra‐tumoral CD8^+^ T cells. Mechanistically, coixenolide inhibited lipid peroxidation and ferroptosis of CD8^+^ T cells, at least in significant part mediated by the S100A8/A9‐CD36 interaction. The potential contribution of Treg modulation warrants further investigation in the future. Our findings identify the S100A8/A9‐CD36 signaling as an immune‐metabolic vulnerability in obesity‐associated PAAD and suggest its therapeutic targeting as a potential strategy to restore CD8^+^ T cell function against the malignancy.

## 5 Materials and Methods

### 5.1 Preparation of Coixenolide‐Loaded Injection

Coixenolide was procured from Zhejiang Kanglaite Pharmaceutical CO., Ltd. (Zhejiang, China). The coixenolide‐loaded injection was formulated following the emulsion preparation guidelines stipulated in the Chinese Pharmacopoeia (2020 edition). Briefly, the aqueous phase was formulated by homogenizing 15 g soybean lecithin and 25 g glycerol (USP grade) in water for injection under aseptic conditions. Simultaneously, 100 g pharmaceutical‐grade coixenolide (purity > 98% by HPLC) was heated to 60°C–70°C and gradually incorporated into the pre‐warmed (60°C–70°C) aqueous phase. The combined system underwent high‐pressure homogenization (EmulsiFlex‐C5, Avestin) at 15 000 psi for 5 cycles to achieve nanoemulsion formation (mean particle size 150 ± 20 nm). The final preparation was adjusted to 1000 mL with WFI, sterile‐filtered (0.22 µm PVDF membrane), and aseptically filled into Type I glass vials. The vehicle control followed identical processing, excluding coixenolide addition.

### 5.2 Sample Collection

This study enrolled four patients with pathologically confirmed pancreatic adenocarcinoma (PAAD). None of the patients had received prior antitumor therapy before undergoing primary PAAD resection at Zhejiang Provincial Hospital of Traditional Chinese Medicine. Surgical intervention was determined following a comprehensive preoperative evaluation, including high‐resolution abdominal magnetic resonance imaging and contrast‐enhanced computed tomography (CT). The cohort comprised both male and female patients, aged 51 to 75 years. Written informed consent for sample collection and data analysis was obtained from all participants prior to surgery. This study was approved by the Ethics Committee of The First Affiliated Hospital of Zhejiang Chinese Medical University (Zhejiang Provincial Hospital of Traditional Chinese Medicine) (Approval number: 2025‐KLS‐283‐01).

### 5.3 Data Collection

The single‐cell RNA sequencing (scRNA‐seq) data were obtained from the Genome Sequence Archive (GSA) database (CRA001160), which included clinical samples from 11 normal individuals and 24 pancreatic adenocarcinoma (PAAD) patients. Additionally, the TCGA‐PAAD cohort was analyzed using the Gene Expression Profiling Interactive Analysis (GEPIA) online tool.

### 5.4 Cell Culture

Pan02 murine pancreatic adenocarcinoma cells and Luciferase‐expressing (Pan02‐luc) cells were purchased from Mingzhou Biotechnology Corporation (Mingzhou, China). *S100a9* knock‐out (KO) Pan02 cells were generated as previously described [32] and were designed and synthesized by RiboBio Biotechnology Co., LTD (Guangzhou, China). All cells were cultured in complete DMEM (GE Healthcare Life Sciences, USA) supplemented with 10% fetal bovine serum (FBS; Gibco; Thermo Fisher Scientific, USA) and 1% penicillin/streptomycin (Yeason Biotechnology, China) at 37°C in a 5% CO_2_ humidified incubator. The Pan02 cell line used in this study has the Research Resource Identifier (RRID): CVCL_D627. The cell line was contamination‐free and underwent Short Tandem Repeat (STR) profiling to confirm its identity.

### 5.5 Mice, Dietary Interventions, and Assessment of the HFD Phenotype

All animal studies were approved by the Ethics Committee and performed in accordance with ethical standards. Five‐week‐old male wildtype C57BL/6J mice (BK, China) were used and assigned to a control diet (CD) with 10% kcal from fat or a high‐fat diet (HFD) with 60% kcal from fat [17], and were fed for a duration of 8 weeks. Body weight was recorded at the indicated experimental time points. After the dietary intervention, the obese/metabolic phenotype was evaluated by gross dorsal anatomical examination and by measurement of blood glucose, cholesterol, and triglyceride levels. All experiments followed the guidelines for the care and use of animals established by the Laboratory Animal Centre, Zhejiang Chinese Medical University (Zhejiang, China) (approval number: IACUC‐20200629‐05).

### 5.6 Tumor Model

An in situ tumor transplantation model was established to evaluate the efficacy and mechanism of coixenolide on tumor suppression in vivo. Briefly, Pan02 cells were suspended in 100 µL normal saline, and then subcutaneously injected into the left or right flank of the mice. When the subcutaneous tumors reached 1 cm^3^ in size, non‐necrotic tumor tissue was transplanted into the pancreas of CD‐ or HFD‐fed mice to establish an in situ tumor transplantation model. The mice with tumors were randomly assigned to one of 6 groups after 7 days and were administered intraperitoneally (i.p.) with coixenolide (1.25 or 2.50 g/kg) or vehicle control once daily for 14 days. During the treatment period, bioluminescence imaging (BLI) was performed using the Small Animal Live Imaging System (IVIS Imaging System; Xenogen Corporation, Alameda, CA, USA). The BLI signals for each mouse were averaged based on luminous area and exposure time, resulting in the generation of BLI_rel luminescence curves. Subsequently, mice were euthanized, and tumor tissues were obtained and fixed in 4% formaldehyde to enable Hematoxylin and Eosin (H&E) staining. Images were acquired by a brightfield light microscope. To verify the in vivo regulation of CD8^+^ T cells by *S100a8/a9*, *S100a9* KO Pan02 cells were used.

### 5.7 Bulk RNA‐Sequencing and Analysis

Tumor tissues from CD and HFD mice, with or without coixenolide treatment, were collected for RNA sequencing analysis. Total RNA was isolated with TRIzol reagent (Invitrogen Corporation) according to the manufacturer's instructions and quantified with a NanoDrop ND‐1000 Spectrophotometer (NanoDrop Technologies, LLC, Wilmington, DE, USA). Dynabeads Oligo (dT) 25‐61005 (Thermo Fisher Scientific) was used to isolate mRNA from total RNA. The cleaved RNA fragments were reverse transcribed into cDNA with SuperScript II Reverse Transcriptase (Invitrogen Corporation). Paired‐end sequencing was performed with a NovaSeq 6000 Sequencing Platform (Illumina, Inc., San Diego, CA, USA) following the vendor's recommended protocol by LC‐Bio Technology Co., Ltd. (Hangzhou, China).

All bioinformatic analyses were performed with R (v4.4.1). Differentially expressed genes (DEGs) were identified with the limma package (version 3.60.6). Gene Ontology (GO) enrichment analysis was performed using the clusterProfiler package (version 4.12.6). Immune infiltration analysis was performed using the Tumor Immune Estimation Resource (TIMER) web tool. Heatmaps were generated using the pheatmap package (version 1.0.12) and volcano plots were generated using the ggVolcano package (version 0.0.2).

### 5.8 Single‐Cell RNA‐Sequencing and Analysis

ScRNA‐seq was conducted by Singleron Biotechnologies (Shanghai, China). In brief, cells were isolated from tumor tissues using GEXSCOPE Nuclear Extraction Buffer (adding RNase inhibitor and DTT before use; the final concentrations of the two are 0.2 U/µL and 1 mm, respectively). Single‐cell suspensions at a concentration of 1 × 10^5^ cells/mL in PBS were prepared. Single‐cell suspensions were then loaded onto microfluidic devices, and scRNA‐seq libraries were constructed according to the Singleron GEXSCOPE protocol using GEXSCOPE Single‐Cell RNA Library Kit (Singleron Biotechnologies), which included cell lysis, mRNA trapping, labeling cells (barcode) and mRNA (UMI), mRNA reverse transcription, cDNA amplification, and finally cDNA fragmentation. Individual libraries were diluted to 4 nm and pooled for sequencing. Pools were sequenced on an Illumina HiSeq X with 150 bp paired‐end reads. Raw reads were processed with fastQC and fastp to remove low‐quality reads. Poly‐A tails and adaptor sequences were removed by cutadapt. Reads were mapped to the reference genome GRCm38 (Ensembl version 92 annotation) using STAR.

Data pre‐processing, normalization, integration, and clustering were performed using the R package Seurat (version 5.1.0). After merging all samples into a single Seurat object, single‐cell transcriptomes were initially filtered using four metrics for quality control. First, cells in which fewer than 400 genes or more than 2500 genes were detected were removed from the analysis. Second, cells where mitochondrial‐encoded transcripts represented greater than 10% of the total library were excluded. Third, cells with more than 12 000 RNA molecules detected per cell were discarded. Finally, genes detected in fewer than three cells across the dataset were removed. Normalization and variance stabilization were performed using the R package harmony (version 1.2.1), which interfaces directly with Seurat, on each diet condition separately. The integrated data were subjected to a principal component analysis (PCA) algorithm to reduce the data dimensions. FindNeighbors and FindClusters were used to cluster cells with similar characteristics. Uniform manifold approximation and projection (UMAP) and t‐distributed stochastic neighbor embedding (t‐SNE) algorithms were used for data visualization.

### 5.9 Differential Expression Analysis, Cell Annotations, and Enrichment Analysis

The default Wilcoxon rank‐sum test was used by running the FindAllMarkers function in Seurat to find DEGs in each cell cluster. Annotation of the resulting clusters to cell types was based on the expression of marker genes. Next, we filtered T cell and epithelial cell subclustering for further analysis. The FindVariableGenes function in Seurat was used to find DEGs between the coixenolide‐treated group and the control group. We set *p*‐value < 0.05, |avg_logFC| > 0.25 as the cutoff criteria. The top 100 genes were filtered based on fold change to perform gene ontology (GO) enrichment analysis using the R package clusterProfiler. The results of the enrichment analysis were selected based on the statistical threshold (cutoff of *p* < 0.05), and the results belonging to biological process (BP) were reserved.

### 5.10 Pseudo‐Time Analysis

CD8^+^ T cell developmental trajectories were inferred using Monocle2 (version 2.99.3) [65] with default parameters as recommended by the developers. First, integrated gene expression matrices from CD8^+^ T cells were exported from Seurat into Monocle to construct a CellDataSet. Second, the ‘setOrderingFilter’ function was applied to sort cells with the variable genes identified by the function of ‘differentialGeneTest’ (cutoff of *q* < 0.001). Finally, after dimensionality reduction using the ‘reduceDimension’ function (using the ‘DDRTree’ reduction method), a series of representative key role genes were revealed along the differentiation progress by the ‘plot_pseudotime‐heatmap’ function. Dimensionality reduction was performed with no normalization and the ‘DDRTree’ reduction method in the ‘reduceDimension’ step. The visualization function “plot_cell_trajectory” was used to plot each group along the same pseudotime trajectory.

In addition, the R package CytoTRACE (version 0.3.3) [66] was applied to calculate the CytoTRACE scores for malignant cells. Higher single‐cell gene counts correlated with less cellular differentiation (higher stemness). CytoTRACE scores ranged from 0 to 1, while higher scores indicated higher stemness (less differentiation) and vice versa.

### 5.11 Malignancy Score Estimation of Epithelial Cells

The scCancer package (version 2.2.1) was used to infer the malignancy score of epithelial cells and to recognize cancer cells with default parameters. We selected normal microenvironment cells (T cells) as the default reference dataset. By comparing the distribution of malignancy scores with the reference and detecting its bimodality, we assigned malignancy labels for cells.

### 5.12 Cell–Cell Interaction Analysis

The CellChat (version 1.1.3) method was used to infer cell‐to‐cell interactions between cancer cells and T cells and to build a regulatory network based on ligand–receptor crosstalk. We filtered out cell–cell communications that were present in fewer than 10 cells in certain cell groups. The netVisual function was used to visualize interaction patterns. When signaling pathways with more than one ligand–receptor pair were evaluated, we used the netAnalysis_contribution function to compute and visualize the contribution of each ligand–receptor pair to the overall signaling pathway. Non‐negative matrix factorization (NMF) algorithms were considered when selecting the number of co‐communication patterns in the CellChat object. This was after utilizing the identifyCommunicationPatterns function, which was adopted to identify major signals for certain cell populations and general communication patterns. In addition, netAnalysis_teCentrality was used to calculate network centrality scores, and netAnalysis_signalingRole_network was used for visualization. These were used to identify dominant senders, receivers, mediators, and influencers in certain inferred networks.

### 5.13 Immunohistochemical Staining

Formalin‐fixed, paraffin‐embedded PAAD tumor tissues from the control group and the 2.5 g/kg coixenolide administration group were collected separately for immunohistochemical (IHC) staining of TUNEL and Ki‐67. The sections were rehydrated and heated in a 10 mmol/L citrate solution (pH 6) for antigen retrieval, followed by treatment with 3% H_2_O_2_/MeOH to suppress endogenous peroxidase activity. The sections were treated with a 10% horse serum blocking solution to prevent nonspecific binding. Primary antibodies were applied to the sections, which were then incubated at 4°C for 16 h in a humidified chamber. Subsequently, the sections were treated with a biotin‐conjugated goat anti‐rabbit IgG secondary antibody (1:2000, ab205718, Abcam). The sections were washed three times with PBS to remove unbound antibodies. Visualization was achieved with a DAB substrate kit. Finally, the slides were digitally scanned with the Pannoramic SCAN II system. The primary antibody used in this study were TUNEL (G1507‐100T, Servicebio) and Ki‐67 (1:2000, GB121141, Servicebio)

### 5.14 Multiplexed Immunofluorescence (IF) Staining

Formalin‐fixed, paraffin‐embedded pancreatic adenocarcinoma (PAAD) tumor tissues from the control group and the 2.5 g/kg coixenolide administration group were collected separately for multiplexed immunofluorescence (IF) staining. Additionally, formalin‐fixed, paraffin‐embedded PAAD specimens from patients were obtained from Zhejiang Provincial Hospital of Traditional Chinese Medicine. Multiple primary antibodies, including Insulin (1:5000, A19066, Abclone), CD3 (1:2000, AB237721, Abcam), CD8 (1:4000, ab237709, Abcam), CD36 (1:200, A26251, Abcam), Pan Cytokeratin (1:2000, 4545T, cst), S100A8/A9 (1:5000, ab288715, Abcam), CD4 (1:500, 67786‐1‐Ig, Proteintech), FOXP3 (1:500, ab20034, Abcam), F4/80 (1:50, ab300421, Abcam), Ly6G (1:200, ab238132, Abcam), GPX4 (1:400, ab125066, Abcam), and 4‐HNE (1:50, MA5‐27570, Thermo Fisher) were used in this study, followed by incubation with horseradish peroxidase‐conjugated secondary antibodies and tyramide signal amplification. The slides were microwave heat‐treated after each tyramide signal amplification operation. Nuclei were stained with DAPI after all the antigens above had been labeled. The stained slides were imaged at ×10 magnification using confocal fluorescence microscopy (DMi8, Leica, Germany), and regions of interest were selected for multispectral image acquisition at ×20 or ×40.

### 5.15 Preparation of Tumor Single‐Cell Suspensions and Flow Cytometry

Fresh tumor tissues were processed into single‐cell suspensions for flow‐cytometric analysis of tumor‐infiltrating lymphocytes and functional markers. For surface‐marker staining, cells were incubated with the indicated fluorophore‐conjugated antibodies after Fc‐receptor blocking; for intracellular cytokines and transcription factors, cells were fixed and permeabilized before staining. Singlets and viable cells were selected before analysis, and CD8^+^ T cells were identified within the lymphocyte/T‐cell gate. Intracellular GZMB (GRB17, Thermo Fisher), TNF‐α (506 306, BioLegend), IFN‐γ (505 808, BioLegend), IL‐2 (503 808, BioLegend), perforin (154 303, BioLegend), and CTLA‐4 (106 308, BioLegend) and surface CD36 (102 708, BioLegend), TLR4 (62‐9041‐80, Thermo Fisher), RAGE (sc‐365154, Santa Cruz), and *S100a8/a9* (ab288715, Abcam) were quantified as required by the corresponding experiments.

### 5.16 Western Blot Analysis

The total protein was extracted by total‐DNA‐RNA‐Protein Kit according to the manufacturer's instructions and separated by electrophoresis on 10% SDS‐PAGE gels, followed by transfer to PVDF membrane. After blocking with 5% BSA, the blots were incubated overnight at 4°C with primary antibodies against *S100a8/a9* (1:100, 765504, BioLegend), *Igkc* (1:500, ab190484, Abcam), *Ighg2c* (1:2000, ab97245, Abcam), *Cyp2e1* (1:2500, ab28146, Abcam), and *Cd36* (1:1000, ab133625, Abcam). Subsequently, the blots were incubated with the horseradish peroxidase‐conjugated secondary antibodies for 2 h, followed by visualization using an enhanced chemiluminescence (ECL, EpiZyme).

### 5.17 Quantitative Real‐Time PCR

Total RNA was extracted from cells using TRIzol reagent (15596018, Invitrogen) and reverse‐transcribed into cDNA. The PCR mixture was prepared containing cDNA, specific primers, and 2× SYBR Green PCR Master Mix (A4004M, Lifeint). Real‐time PCR was performed on a CFX96 Touch Real‐Time PCR Detection System (Bio‐Rad) under the following conditions: initial denaturation at 95°C for 10 min, followed by 40 cycles of 95°C for 12 s and 60°C for 40 s. The primer sequences used were as follows: *S100a8* forward, CCTGAAGGTTCTGTTTTTCAGGT; *S100a8* reverse, GGTCATCCCTGTAGACGGCA; *S100a9* forward, AGCCTTCGCCATCTTCTAC; *S100a9* reverse, GTCCTCCTTGGCAGTTTCCT; GAPDH forward, AGCTTCAGCCCCAGGAAATC; and GAPDH reverse, GACATACTGCTGGGCCAGTT. GAPDH was used as the housekeeping gene, and relative mRNA expression levels of the target genes were calculated accordingly.

### 5.18 CD8^+^ T Cell Cytotoxicity Assay

To generate mature CD8^+^ T cells, splenocytes were isolated from C57BL/6J mice using a mouse spleen lymphocyte isolation kit (PLA2011M, TBD). CD8^+^ T cells were then purified by anti‐mouse CD8a particles (551516, BD Pharmingen) and cultured in RPMI 1640 medium containing 5 µg/mL anti‐mouse CD3e (553057, BD Pharmingen), 1 µg/mL anti‐mouse CD28 (553294, BD Pharmingen), 10 ng/mL murine IL‐2 (550069, BD Pharmingen), 10% FBS, and 1% penicillin‐streptomycin. To measure the cytotoxicity of CD8^+^ T cells, 250 000 enriched CD8^+^ T cells were mixed in 6‐well plates with Pan02 cells at ratios of 5:1.

The killing efficiency of CD8^+^ T cells was assessed using flow cytometry 24 h after co‐culture with tumor cells. Briefly, Pan02 cells or CD8^+^ T cells were pretreated with or without coixenolide for 24 h. Subsequently, the suspended CD8^+^ T cells were collected, and the adherent Pan02 cells at the bottom were harvested after digestion. The Pan02 cells were stained with PI using an Annexin V‐FITC/PI Apoptosis Detection Kit (BL110, Yeason Biotechnology) to measure the percentage of apoptotic cells on a flow cytometer. The CD8^+^ T cell supernatants were collected, and the secretion of TNF‐α (Mouse TNF‐alpha ELISA Kit, RK00027, Abclonal), IFN‐γ (Mouse IFN‐gamma ELISA Kit, RK00019, Abclonal), and GZMB (Mouse Granzymes B ELISA Kit, RK00007, Abclonal) was quantified using specific ELISA kits following the standard protocols. The CD8^+^ T cells were stained with Carboxyfluorescein succinimidyl ester (CFSE, 565082, BD Pharmingen) to measure cell proliferation on a flow cytometer. For the S100A8/A9 treatment assay, Pan02 cells as well as CD8^+^ T cells were co‐cultured with recombinant mouse S100A8/A9 heterodimer (20 µg/mL, BioLegend). Pan02 cells were pretreated with tasquinimod (125 µm, ABR‐215050, MCE) before co‐culture with activated CD8^+^ T cells.

### 5.19 Isolation and Culture of Regulatory T (Treg) Cells

To generate and culture Treg cells, splenocytes were first isolated from C57BL/6J mice. CD4^+^CD25^+^ Treg cells were subsequently purified using a CD4^+^CD25^+^ Treg cell isolation kit (130‐092‐984, Univ) according to the manufacturer's instructions. The purified Treg cells were cultured in RPMI 1640 medium supplemented with 5 µg/mL anti‐mouse CD3e, 1 µg/mL anti‐mouse CD28, 10 ng/mL murine IL‐2, 5 ng/mL murine TGF‐β1 (7666‐MB‐005, R&D Systems), 10% fetal bovine serum (FBS), and 1% penicillin‐streptomycin. The culture medium was replaced every 2,3 days, and cells were maintained at 37°C in a humidified atmosphere containing 5% CO_2_ for 5–7 days to obtain viable and functional Treg cells. Following treatment with coixenolide for 24 h, Treg cells were stained with PE‐conjugated anti‐mouse CTLA‐4 antibody (A27774, ABclonal) and FITC‐conjugated anti‐mouse IL‐10 antibody (A27680, ABclonal), and the expression levels of CTLA‐4 and IL‐10 were analyzed by flow cytometry. To assess the regulatory effect of Treg cells on CD8^+^ T cells, Treg cells were co‐cultured with CD8^+^ T cells at a 1:1 ratio.

### 5.20 Lipid Peroxidation and Cytosolic ROS Measurement

Experiments were performed according to the manufacturer's protocol. Briefly, CD8^+^ T cells were collected and incubated in a humidified chamber at 37°C with 5% CO_2_ for 30 min with Lipid Peroxidation Sensor (ab243377, Abcam) or CM‐H_2_DCFDA (C6827, ThermoFisher) in cell culture medium. After incubation, cells were washed and examined by flow cytometry within 2 h of staining.

### 5.21 Cellular Iron Staining

CD8^+^ T cells were washed with PBS twice and stained with 10 nm of Phen Green SK (P14313, ThermoFisher) for 15 min in 37°C. After staining, cells were washed and examined by flow cytometry within 2 h of staining.

### 5.22 Fatty Acid Uptake Assay

The fatty acid uptake kit (MAK156, Sigma‐Aldrich) was utilized to determine the free fatty acid uptake in CD8^+^ T cells. In brief, CD8^+^ T cells isolated from each group were incubated with a fluorescent fatty acid probe (37°C), followed by quantification of fluorescence intensity via a microplate reader at excitation/emission wavelengths of 488/523 nm.

### 5.23 Co‐Immunoprecipitation of the S100A8/A9‐CD36 Complex

Co‐immunoprecipitation was performed on lysates from activated CD8^+^ T cell‐Pan02 cocultures with or without coixenolide treatment to assess physical association between *Cd36* and *S100a8/a9*. Equal amounts of protein were subjected to immunoprecipitation with an antibody against *Cd36* (or the reciprocal *S100a8/a9* antibody) and an isotype IgG control, followed by protein A/G bead capture, washing, elution, SDS‐PAGE, and immunoblotting for *Cd36* and *S100a8/a9*.

### 5.24 TLR4 and RAGE Activation Assays

To evaluate whether activation of TLR4 or RAGE signaling altered the effect of coixenolide, activated CD8^+^ T cells were cocultured with Pan02 cells, and the coculture was treated with control, pathway agonist alone, coixenolide alone, and agonist plus coixenolide. Lipopolysaccharide (LPS, 100 ng/mL) was used to activate TLR4 signaling, whereas advanced glycation end products (AGEs, 100 µg/mL) were used to activate RAGE signaling. Pan02 cells were pretreated with or without coixenolide using the same tumor‐cell pretreatment scheme as described for the coculture experiments. LPS or AGEs were added during the co‐culture period. After treatment, TNF‐α production by CD8^+^ T cells was quantified by flow cytometry.

### 5.25 Generation and Analysis of Cd36‐Knockout CD8^+^ T Cells

To genetically test CD36 dependence, primary CD8^+^ T cells were subjected to CRISPR‐Cas9‐mediated *Cd36* knockout before co‐culture experiments [39]. WT and *Cd36*‐KO CD8^+^ T cells were compared with or without coixenolide exposure, and lipid peroxidation, cell death, cellular iron, cytosolic ROS, and fatty‐acid uptake were measured.

### 5.26 PD‐1 Blockade in the CD8^+^ T Cell‐Pan02 Coculture Model

To explore potential combination activity with immune‐checkpoint blockade, activated CD8^+^ T cells were cocultured with Pan02 cells in the presence or absence of coixenolide and anti‐PD‐1 antibody clone 29F.1A12. CD8^+^ T‐cell cytotoxic activity and tumor‐cell killing were then evaluated. Expression of CD274 (PD‐L1) in tumor cells and PDCD1 (PD‐1) in CD8^+^ T cells was analyzed in the corresponding bioinformatic/coculture comparisons.

### 5.27 Statistical Analysis

Continuous data were first assessed for normality using the Shapiro–Wilk test and for homogeneity of variances using Levene's test. Outliers were identified by the ROUT method (*Q* = 1%) and were excluded only if a technical artifact was confirmed; otherwise, all data points were retained. No data transformation or normalization was applied except where noted (e.g., gene expression values were log2‐transformed for heatmap visualization). Data are presented as mean ± standard deviation (SD) from at least three independent biological replicates, unless otherwise specified. The exact sample size (*n*) for each experimental condition is provided in the figure legends or results text (e.g., *n* = 3 independent cell culture replicates). For comparisons between two groups, a two‐sided Student's *t*‐test was used for normally distributed data; otherwise, the Mann–Whitney *U*‐test (Wilcoxon rank‐sum test) was applied. For comparisons involving more than two groups, one‐way analysis of variance (ANOVA) followed by Tukey's post‐hoc test for multiple comparisons was performed when the assumptions of normality and homogeneity of variances were met. If these assumptions were violated, the Kruskal–Wallis test with Dunn's post‐hoc correction was used. All hypothesis tests were two‐sided with a pre‐defined significance level (alpha) of 0.05. Adjusted *P*‐values are reported for multiple comparisons. No additional alpha adjustment (e.g., Bonferroni) was applied beyond the post‐hoc corrections described, as these were considered appropriate for the pairwise comparisons performed. The validity of test assumptions (normality, homoscedasticity) was verified for each analysis, and any deviation is noted in the corresponding figure legend. Statistical analyses were performed using GraphPad Prism software (version 9.0).

## Author Contributions

Mancang Gu, Lei Huang, Shanming Ruan, and Zhaoshen Li conceived and designed the study. Kaidi Chen, Rong Chen, Wei Li, Bin Song, Ruiying Zhang, Lewei Dai, Yuwei Qi, Ruqian Chen, Yifan Xu, Hangbin Li, Jingyi Huang, Fangping Wu, and Jianan Peng collected samples and performed the experiments. Kaidi Chen, Rong Chen, Wei Li, Bin Song, Lei Huang, and Mancang Gu conducted the data analysis. Kaidi Chen, Rong Chen, Wei Li, Lei Huang, and Mancang Gu drafted the manuscript. All authors reviewed and revised the manuscript.

## Funding

This work was supported by National Natural Science Foundation of China (82474338, 82274364, 81774011, and 82230018), Zhejiang Province Natural Science Foundation (LZ23H290001 and LY19H280001), Zhejiang Provincial “Three Rural Areas and Nine Parties Agricultural Science and Technology Collaborator Program” (2025SNJF075), the General Scientific Research Project of Zhejiang Provincial Education Department (Y202456442 and 2025YKJ10), the Scientific Research Start‐up Fund for Excellent Doctor (Youbo; Class A; L.H.) (The Third Batch) by The First Affiliated Hospital of Naval Medical University/Changhai Hospital, Naval Medical University, the Xueshu Xinxiu Talent Fund (L.H.) by Naval Medical University, and the Shenlan Qihang Talent Fund (The Third Batch; L.H.) by Naval Medical University. The funders played no roles in study design; in the collection, analysis, or interpretation of data; in the writing of the report; or in the decision to submit the paper for publication.

## Conflicts of Interest

The authors declare no conflicts of interest.

## Supporting information




**Supporting File**: advs77292‐sup‐0001‐SuppMat.docx.

## Data Availability

Data are available upon reasonable request from the authors with permissions from the institutions.
