# Supplementary material for: Coixenolide Enhances Antitumor Immunity of Cytotoxic CD8+ T Cells by Inhibiting S100A8/A9‐CD36 Axis in Obesity‐Associated Pancreatic Adenocarcinoma
Source: Adv Sci (Weinh). 2026 Aug 25:e15759. Online ahead of print. doi: 10.1002/advs.202515759 (PMC13505587; doi:10.1002/advs.202515759)
Supplement: Supplementary file 1 [file ADVS-9999-e15759-s001.docx]

**Supplementary Materials**

**Figures S1-S9**

**Figure S1. *In vivo* pharmacodynamic study of coixenolide against PAAD.** (**A-D**) The body weight (**A**), blood glucose levels (**B**), cholesterol levels (**C**), and triglyceride levels (**D**) of CD- or HFD-fed mice. (**E and F**) Gross images of excised tumors from CD-fed (**E**) and HFD-fed (**F**) mice after 14 days of vehicle or coixenolide treatment. n = 5 per group. (**G**) Oil Red O staining results revealed the extent of adipocyte infiltration in tumors from CD- or HFD- fed mice. (**H and I**) The changes in the average body weight of CD (**H**) and HFD (**I**) mice in the coixenolide treatment group compared to the vehicle control group during the treatment period. (**J and K**) DEGs in HFD (**J**) and CD (**K**) tumors following coixenolide treatment (2.5g/kg). (**L**) A Venn diagram was used to compare differentially expressed genes (DEGs) between CD and HFD tumor groups after coixenolide administration. (**M and N**) The immune cell proportions in HFD (**M**) or CD (**N**) tumors between coixenolide treated and control groups using CIBERSORT, CIBERSORT absolute mode (CIBERSORT-ABS), EPIC, MCPCOUNTER, QUANTISEQ, TIMER and XCELL. Red stars denote elevated immune cell proportions, while blue stars indicate reduced proportions in the coixenolide-treated group, respectively. **p* < 0.05, ***p* < 0.01, ****p* < 0.001.

**Figure S2.** **Heterogeneous tumor microenvironment remodeling in mice treated with coixenolide.** (**A and B**) T cell subtype proportion of control group and coixenolide treated group within the tumor microenvironment of HFD-fed (**A**) and CD-fed (**B**) mice. (**C**) Immunofluorescent staining showing co-localization of CD4 (orange), FOXP3 (wheat), and DAPI (deep blue) in and HFD tissues following coixenolide administration. Scale bars, 100 μm. (**D**) Distribution of cytotoxic CD8^+^ T cells in the tissues of CD- and HFD-fed mice with or without coixenolide treatment. (**E**) The pathways enrichment associated with the top 100 upregulated genes (ranked by *p* value) in the tumors of HFD-fed mice following coixenolide administration. (**F**) Gating strategy used for flow cytometric detection of effector function in tumor-infiltrating CD8⁺ T cells isolated from tumor-bearing mice with or without coixenolide treatment. ***p* < 0.01.

**Figure S3.** **Clinical cohort analysis confirmed significant overexpression of S100A8/A9 in tumor cells.** (**A**) Histogram shows the distribution of the estimated malignancy scores of observation (blue) of reference (gray) cells. (**B**) The CytoTRACE score of each epithelial cell subcluster. (**C**) The expression of *S100a8/a9*. Cancer cells with the highest *S100a8/a9* expression level are colored red. (**D**) Adipocyte-conditioned medium (ACM) upregulated *S100a8/a9* protein expression in murine pancreatic cancer Pan02 cells. (**E**) UMAP plot displaying the integrated cell map, which consists of 10 annotated cell types. (**F**) Expression levels of S100A8 and S100A9 among 10 annotated cell types. (**G**) Expression levels of S100A8 and S100A9 between ductal 1 and ductal 2 cells. (**H**) Quantitative real-time PCR (qPCR) analyses of *S100a8* and *S100a9* mRNA expressions in Pan02 cells treated with or without coixenolide. (**I**) Coixenolide modulated transcriptional regulatory pathways in *S100a8/a9^+^* tumor cells. (**J**) Coixenolide modulated post-translational modification-related pathways in *S100a8/a9^+^* tumor cells. (**K**) Effects of coixenolide treatment on the expressions of *Cyp2e1*, *Ighg2c*, and *Igkc* in Pan02 cells.

**Figure S4. Coixenolide showed low cytotoxicity on both CD8^+^ T and PAAD cells.** (**A**) Outgoing communication patterns of secretory cells. Dot plot showed the contribution of each cell population to ligand secretion, with dot size indicating contribution strength. Red arrows highlight the profiles of *S100a8/a9^+^* tumor cell clusters, cytotoxic CD8⁺ T cells, and Treg cells. (**B**) Cell-cell interaction network analysis of tumor-infiltrating immune cells in the control (left) and coixenolide-treated (right) tumor tissues, showing the number of interactions between tumor cells and immune cell subsets. (**C**) Bubble plot illustrated the incoming and outgoing interaction strengths of immune cell populations and *S100a8/a9*⁺ tumor cells (highlighted in red). (**D**) Quantification of *S100a8/a9*⁺ cells across distinct cell populations in the tumor microenvironment. (**E and F**) Coixenolide was tested for toxicity toward Pan02 cells (**E**) and CD8^+^ T cells (**F**) freshly isolated from C57BL/6 mice. (**G**) Lactate dehydrogenase (LDH) release assay was used to measure the cytotoxicity of activated CD8⁺ T cells against Pan02 tumor cells. (**H**) Flow cytometry with propidium iodide (PI) staining was performed to assess tumor cell viability in the same coculture system. Representative histograms (upper) and quantification of the percentage of PI⁺ tumor cells (lower) are shown. Activated CD8⁺ T cells were treated with or without an anti-CD8α neutralizing antibody before coculture with control or coixenolide-pretreated Pan02 cells. (**I**) Flow cytometric analysis of the mean fluorescence intensity (MFI) of CTLA-4 and IL-10 of Tregs from co-cultures treated with or without coixenolide. (**J**) Effect of coixenolide-pretreated Treg cells on the effector function of CD8⁺ T cells in coculture. **p* < 0.05, ***p* < 0.01, ****p* < 0.001.

**Figure S5. Coixenolide enhanced CD8^+^ T cell function by modulating the interaction between S100A8/A9^+^ PAAD and CD8^+^ T cells.** (**A-C**) The levels of TNF-α (**A**), IFN-γ (**B**) and GZMB (**C**) in the supernatants after coculture of CD8^+^ T cells with Pan02 cells with S100A8/A9 inhibitor tasquinimod (+) or DMSO (-) were determined by ELISA. (**D**) Flow cytometry analyses of T cell exhaustion markers (CTLA-4) in CD8^+^ T cells using coculture of Pan02 cells and CD8^+^ T cells pretreated with tasquinimod (+) orDMSO (-). (**E**) Pan02 cells were transfected with CRISPR‑Cas9‑mediated gene knockout constructs expressing *S100a9* sgRNA (*S100a9*-sgRNA) or empty (control-sgRNA) for 8 h, then the *S100a8/a9* protein expression was determined by western blot analysis. (**F**) Crystal violet staining analysis revealed the proliferative changes in tumor cells following *S100a9* knockout. ****p* < 0.001.

**Figure S6. PAAD cell-derived S100A8/A9 bound to CD36 and mediated lipid peroxidation and ferroptosis in CD8^+^ T cells.** (**A-B**) Representative immunofluorescence images of tumor tissues from mice fed with control diet (CD) or high-fat diet (HFD), and treated with or without coixenolide. Staining: DAPI (blue, nuclei), CD3 (light blue, T cells), CD8 (green, cytotoxic T cells), CD36 (pink), and S100A8/A9 (red). White arrows indicate S100A8/A9-expressing macrophages (F4/80) or neutrophils (Ly6G). Red arrows indicate CD36-expressing CD8⁺ T cells. Scale bars, 200 μm. (**C-D**) Flow cytometric analyses of TLR4 (**C**) and RAGE (**D**) expressions on CD8⁺ T cells cocultured with tumor cells, with or without coixenolide pretreatment of tumor cells. Representative histograms (left) and quantification of mean fluorescence intensity (MFI) (right) are shown. (**E-F**) Flow cytometry analyses of TNF-α production by CD8⁺ T cells cocultured with tumor cells. CD8⁺ T cells were stimulated with LPS (**E**) or AGEs (**F**) to activate TLR4 or RAGE signaling. Tumor cells were pretreated with or without coixenolide. Representative histograms (left) and MFI quantifications (right) are shown. (**G-H**) The change of ferroptosis inducer (**G**) and inhibitor (**H**) related genes after coixenolide administration. (**I-J**) S100A8 and S100A9 among 24 pancreatic tumor samples (**I**) and S100A8/A9 low and high expression groups (**J**). (**K**) The expression of CD8A among 8 T cell subclusters. (**L-M**) Gene set enrichment of lipid peroxidation, ferroptosis (**L**) and lipid transport (**M**) related genes in tumor-infiltrating CD8^+^ T cells. (**N**) Genes associated with lipid peroxidation or ferroptosis expression between S100A8/A9^high^ patients and S100A8/A9^low^ patients. (**O**) Gene set enrichment of regulation of T cell mediated cytotoxicity related genes. (**P**) The expression of CD8^+^T cell genes associated with exhaustion between S100A8/A9 low and high expression groups. (**Q**) CD8⁺ T cells were transfected with a sgRNA targeting *Cd36* (*Cd36*‑sgRNA) for CRISPR‑Cas9‑mediated *Cd36*‑knockout (*Cd36*‑KO) or an empty control sgRNA (control‑sgRNA) after transfection and recovery, then the *Cd36* protein expression was determined by Western blot analysis. (**R-V**) Analyses of lipid peroxidation (**R**), cell death (**S**), iron levels (**T**), cytosolic ROS (**U**), and fatty acid uptake (**V**) in WT and *Cd36* KO CD8⁺ T cells cocultured with Pan02 cells pretreated with or without coixenolide. **p* < 0.05, ***p* < 0.01, ****p* < 0.001.

**Figure S7. Coixenolide inhibits S100A8/A9-CD36-mediated fatty acid uptake to attenuate lipid peroxidation and ferroptosis of CD8⁺ T Cells.** (**A-E**) Flow cytometry analyses of lipid peroxidation (**A**), cell death (**B**), iron (**C**), cytosolic ROS (**D**), and free fatty acid (**E**) of CD8^+^ T cells. CD8⁺ T cells and Pan02 tumor cells were cocultured in medium supplemented with either fetal bovine serum (FBS) or Insulin-Transferrin-Selenium (ITS-G, 100×). ITS-G was used to replaced FBS to establish a fatty acid-deprived coculture environment. (**F-J**) Analyses of lipid peroxidation (**F**), cell death (**G**), iron (**H**), cytosolic ROS (**I**), and free fatty acid (**J**) of CD8^+^ T cells. In the ITS‑G‑supplemented coculture system, tumor cells were pretreated with coixenolide, CD8⁺ T cells were treated with SSO, and/or recombinant S100A8/A9 protein was exogenously added to the coculture as indicated.

**Figure S8. *S100a9* knockout enhances tumor-infiltrating CD8⁺ T-cell abundance and effector activity in CD-fed mice.** (**A**) Flow cytometric analysis of CD8^+^ T cells in *S100a9* WT, KO and coixenolide treated KO tumors. (**B**) The quantification results of CD8^+^ T cell subpopulations isolated from *S100a9* WT, KO and coixenolide treated KO tumors. (**C**) Flow cytometric analysis of CD8^+^ T cell effector molecules including IL-2, GZMB, perforin, and TNF-α in *S100a9* WT, KO and coixenolide treated KO tumors. (**D**) The quantification results of CD8^+^ T cell effector molecules isolated from *S100a9* WT, KO and coixenolide treated KO tumors. **p* < 0.05, ***p* < 0.01, ****p* < 0.001.

**Figure S9.** **Coixenolide treatment downregulates PD-1/PD-L1 expression and enhances the effector function of CD8⁺ T cells in PAAD.** (**A**) Correlation analysis of S100A8/A9 expression with PDCD1 (encoding PD-1) and CD274 (encoding PD-L1). (**B**) Violin plots demonstrating a marked decrease in *Pdcd1* expression in CD8⁺ T cells and *Cd274* expression in tumor cells after coixenolide administration. (**C**) Flow cytometric analysis of PI staining. (**D**) Flow cytometry histogram and quantitative analysis of TNF‑α expression. **p* < 0.05, ***p* < 0.01, ****p* < 0.001.
